# A Complete Electron Microscopy Volume of the Brain of Adult *Drosophila melanogaster*

**DOI:** 10.1016/j.cell.2018.06.019

**Published:** 2018-07-26

**Authors:** Zhihao Zheng, J. Scott Lauritzen, Eric Perlman, Camenzind G. Robinson, Matthew Nichols, Daniel Milkie, Omar Torrens, John Price, Corey B. Fisher, Nadiya Sharifi, Steven A. Calle-Schuler, Lucia Kmecova, Iqbal J. Ali, Bill Karsh, Eric T. Trautman, John A. Bogovic, Philipp Hanslovsky, Gregory S.X.E. Jefferis, Michael Kazhdan, Khaled Khairy, Stephan Saalfeld, Richard D. Fetter, Davi D. Bock

**Affiliations:** 1 Janelia Research Campus, Howard Hughes Medical Institute, Ashburn, VA 20147, USA; 2 Coleman Technologies, Newtown Square, PA 19073, USA; 3 Hudson Price Designs, Hingham, MA 02043, USA; 4 Division of Neurobiology, MRC Laboratory of Molecular Biology, Cambridge CB2 0QH, UK; 5 Department of Zoology, University of Cambridge, Cambridge CB2 3EJ, UK; 6 Department of Computer Science, Johns Hopkins University, Baltimore, MD 21218, USA

**Keywords:** electron microscopy, connectomics, neural circuits, *Drosophila melanogaster*, mushroom body, olfaction, image stitching

## Abstract

*Drosophila melanogaster* has a rich repertoire of innate and learned behaviors. Its 100,000-neuron brain is a large but tractable target for comprehensive neural circuit mapping. Only electron microscopy (EM) enables complete, unbiased mapping of synaptic connectivity; however, the fly brain is too large for conventional EM. We developed a custom high-throughput EM platform and imaged the entire brain of an adult female fly at synaptic resolution. To validate the dataset, we traced brain-spanning circuitry involving the mushroom body (MB), which has been extensively studied for its role in learning. All inputs to Kenyon cells (KCs), the intrinsic neurons of the MB, were mapped, revealing a previously unknown cell type, postsynaptic partners of KC dendrites, and unexpected clustering of olfactory projection neurons. These reconstructions show that this freely available EM volume supports mapping of brain-spanning circuits, which will significantly accelerate *Drosophila* neuroscience.

**Video Abstract:**

## Highlights

• A complete adult *Drosophila* brain was imaged with EM and has been made publicly available
• The imaged volume enables brain-spanning mapping of circuits at synaptic resolution
• All mushroom body (MB) calyx inputs were mapped, revealing a new cell type, MB-CP2
• Previously unidentified synaptic partners form recurrent microcircuits in MB calyx

## Graphical Abstract

**Figure:**
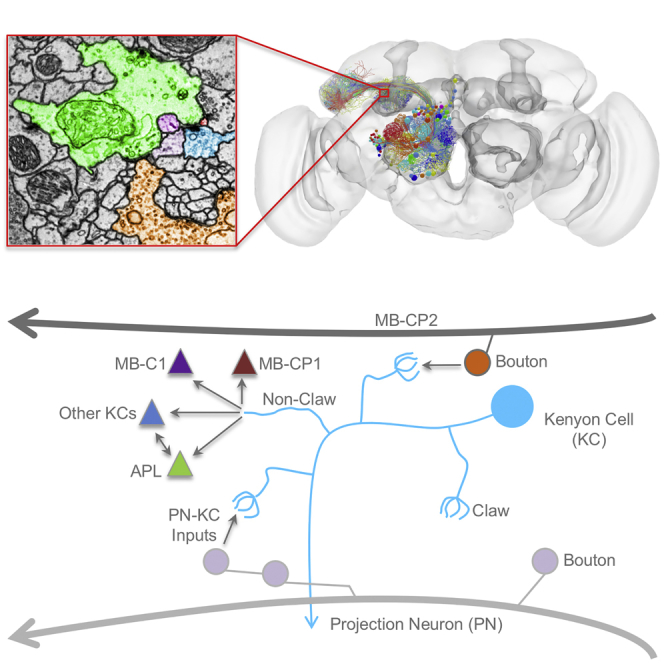

## Teaser

Electron microscopy imaging of the entire adult fruit fly brain at synapse resolution reveals circuitry spanning multiple regions and connectivity between known and previously unknown cell types.

## Introduction

How brain circuits allow animals to implement complex behavior remains a central mystery of neurobiology. Where available, neuronal “wiring diagrams” or “connectomes” (Lichtman and Sanes, 2008)—maps of the synaptic connectivity between the neurons in a circuit—have proved extremely useful for understanding circuit function (Ding et al., 2016, Jarrell et al., 2012, Kasthuri et al., 2015, Ohyama et al., 2015, Takemura et al., 2017b, Wanner et al., 2016). However, many neuronal circuits are brain-spanning, and access to whole-brain connectomes has been limited to a few small organisms, such as the nematode *C. elegans*, the larva of the fruit (or vinegar) fly, *Drosophila melanogaster*, and the tadpole larva of the tunicate *Ciona intestinalis* (Ohyama et al., 2015, Ryan et al., 2016, White et al., 1986).

**Figure 1:**
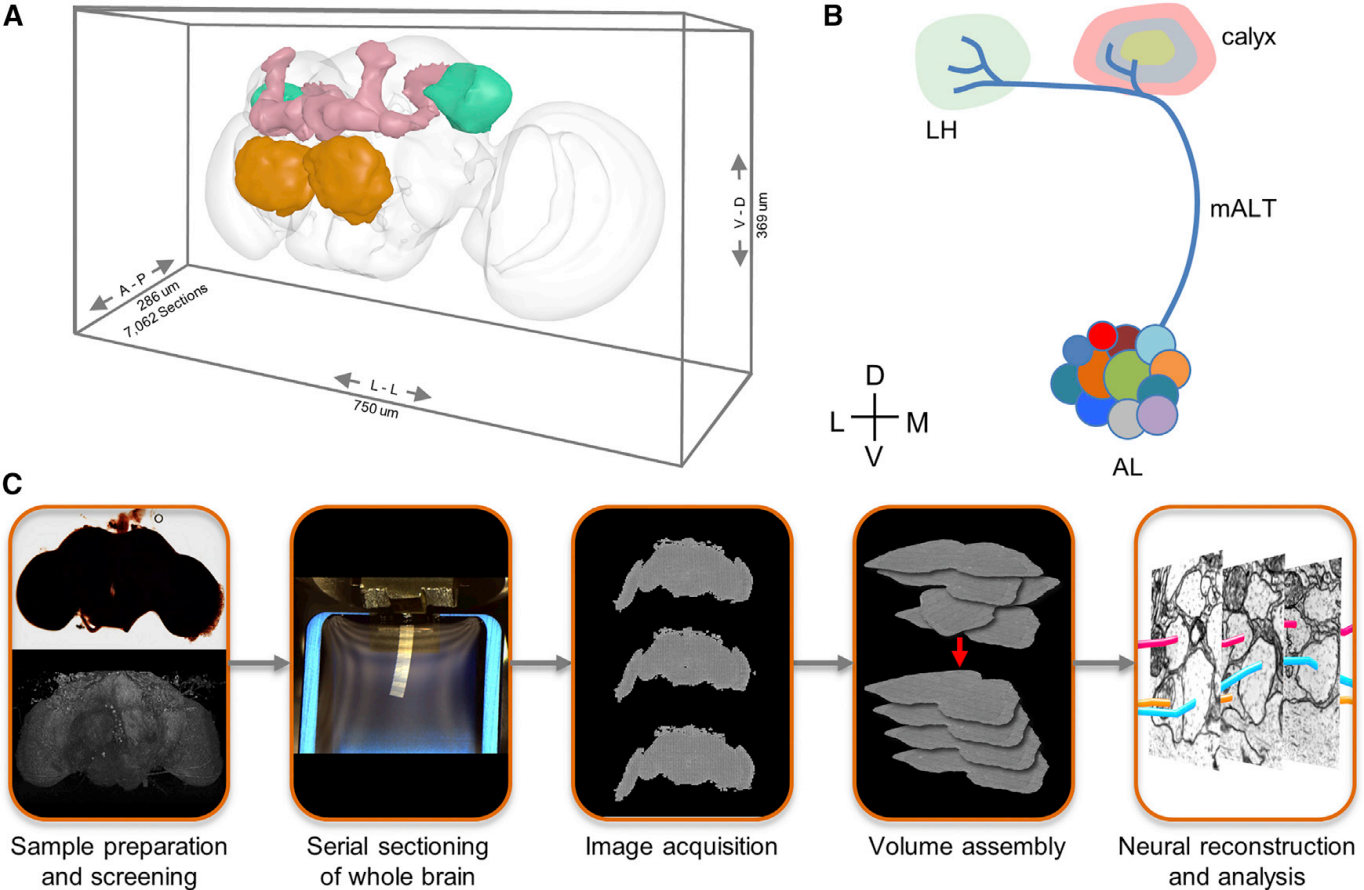
Target Volume and EM Acquisition Infrastructure (A) Oblique view of a surface model of the *Drosophila* brain (gray mesh) with specific neuropil compartments highlighted: AL (orange), MB (pink), and LH (green). (B) Schematic of olfactory pathway. Approximately 150 PNs, divided into ∼50 subtypes based on their anatomically defined glomeruli of origin in the AL, project to the MB calyx and LH (Grabe et al., 2016, Masse et al., 2009). Lateral horn is thought to mediate innate olfactory behaviors, whereas the MB is involved in learned behaviors (Keene and Waddell, 2007). In MB calyx, PN collaterals terminate in boutons and synapse on KCs (Yasuyama et al., 2002). Most PN types project to the MB calyx via the mALT, but several travel in secondary tracts (data not shown), and a few bypass calyx and project only to LH (Frank et al., 2015, Stocker et al., 1990, Tanaka et al., 2012). (C) Workflow for the generation of the whole-brain dataset. Blocks of brain tissue are incubated in heavy metals to label cell membranes, embedded in a resin polymer, and screened with X-ray tomography. Blocks were then serially sectioned with a diamond knife (for an introduction to serial sectioning techniques, see Harris et al., 2006). Groups of three serial sections are placed on metal slot grids for imaging in one of the two custom high-throughput TEM systems (TEMCA2 or ATPS). The imaged sections were assembled into an aligned volume with the custom software pipeline described here. Reconstruction and analyses of neural circuits in the volume were conducted with the CATMAID tracing environment (Saalfeld et al., 2009). D, dorsal; V, ventral; A, anterior; P, posterior; M, medial; L, lateral; LH, lateral horn; mALT, medial antennal lobe tract. See also Figures S1, S2, and S3 and Videos S1, S2, S3, and S4.

The adult fruit fly has emerged as a key genetic model system for interrogating the neuronal substrates of sophisticated behaviors, such as place learning, flight control, courtship, grooming, and memory-driven action selection (Dickinson and Muijres, 2016, Hampel et al., 2015, Ofstad et al., 2011, Owald and Waddell, 2015, Pavlou and Goodwin, 2013). Given the morphological and physiological stereotypy and genetic accessibility of neuronal cell types in the fly brain, connectomes of circuits underlying these behaviors should translate well across individuals and significantly accelerate the dissection of the neuronal basis for behavior (Gruntman et al., 2018, Jovanic et al., 2016, Ohyama et al., 2015, Takemura et al., 2017a, Takemura et al., 2017b). However, at ∼8 × 10^7^ μm^3^ and ∼100,000 neurons (Simpson, 2009), the brain of an adult fly is two orders of magnitude larger than that of the fruit fly larva, the next-largest brain imaged at synaptic resolution (Ohyama et al., 2015). This combination of scale and resolution has heretofore been unattainable by volume electron microscopy (EM), the only method capable of simultaneously resolving all neuronal branches and synapses in a given volume of brain tissue (Helmstaedter et al., 2008). Therefore, we built new hardware and software for high-speed acquisition and processing of serial section transmission EM (TEM) images and used this infrastructure to image a whole-fly brain at synaptic resolution (Figure 1).

We took multiple approaches to validate our whole-brain volume for tracing synaptic connectivity of brain-spanning neuronal circuits. Our efforts focused on the mushroom body (MB), which has been intensively studied for its role in associative memory formation and recall (Guven-Ozkan and Davis, 2014, Keene and Waddell, 2007). Olfactory projection neurons (PNs) provide the main sensory input to the MB; their connections with the MB intrinsic neurons, Kenyon cells (KCs), form a crucial stage of a fan-out fan-in network analogous to brain structures including the mammalian cerebellum (Farris, 2011, Stevens, 2015). Furthermore the logic of PN to KC connectivity ratios has been the subject of detailed experimental and theoretical analysis as a model for the construction of high dimensional sensory representations (e.g., Caron et al., 2013, Litwin-Kumar et al., 2017). KCs and PNs are brain spanning, morphologically stereotyped, and anatomically extremely well described at the light level (Aso et al., 2014, Jefferis et al., 2007, Tanaka et al., 2012), making them well suited for validating the accuracy of neural reconstructions in the volume. We have developed software tools to enable co-visualization, quantitative analysis, and rapid cell type identification by merging EM reconstructions with existing large-scale light microscopy (LM) databases of neuronal morphology (Chiang et al., 2011, Costa et al., 2016, Milyaev et al., 2012).

Independent tracing of Kenyon cell dendrites, which have some of the finest neurites in the fly brain (Yasuyama et al., 2002), provided a sensitive test of the consistency of neural reconstructions. Retrograde tracing from KC dendrites provided a complete enumeration of olfactory PN input to the MB and yielded an improved map of local circuitry in the calyx, the initial site for sampling and processing of sensory information in the MB. This revealed principles of coordinated organization that were invisible in previous work using light level data assembled from many different brains; for example we found a high degree of clustering of PN inputs, which may generate biases in PN-to-KC connectivity and therefore shape how olfactory PN input to the MB is sampled. Although the MB has been intensively studied, we also discovered a previously unknown, brain-spanning neuron that provides input to KCs and likely relays non-olfactory, multimodal information to the calyx. Finally, we show that KC dendrites make output synapses onto a small subset of available cell types, defining a specific local recurrent microcircuit in the calyx.

In conclusion, we describe the largest synaptic-resolution, whole-brain EM dataset obtained to date. We show that it enables efficient mapping and identification of both known and unknown neurons in adult *Drosophila*, a key model system for circuit neuroscience and, crucially, that it enables reliable and efficient determination of synaptic connectivity. We have made these data and supporting software freely available for download and immediate use by the scientific community.

## Results

### New Tools for Volume EM Data Acquisition

**Figure S3:**
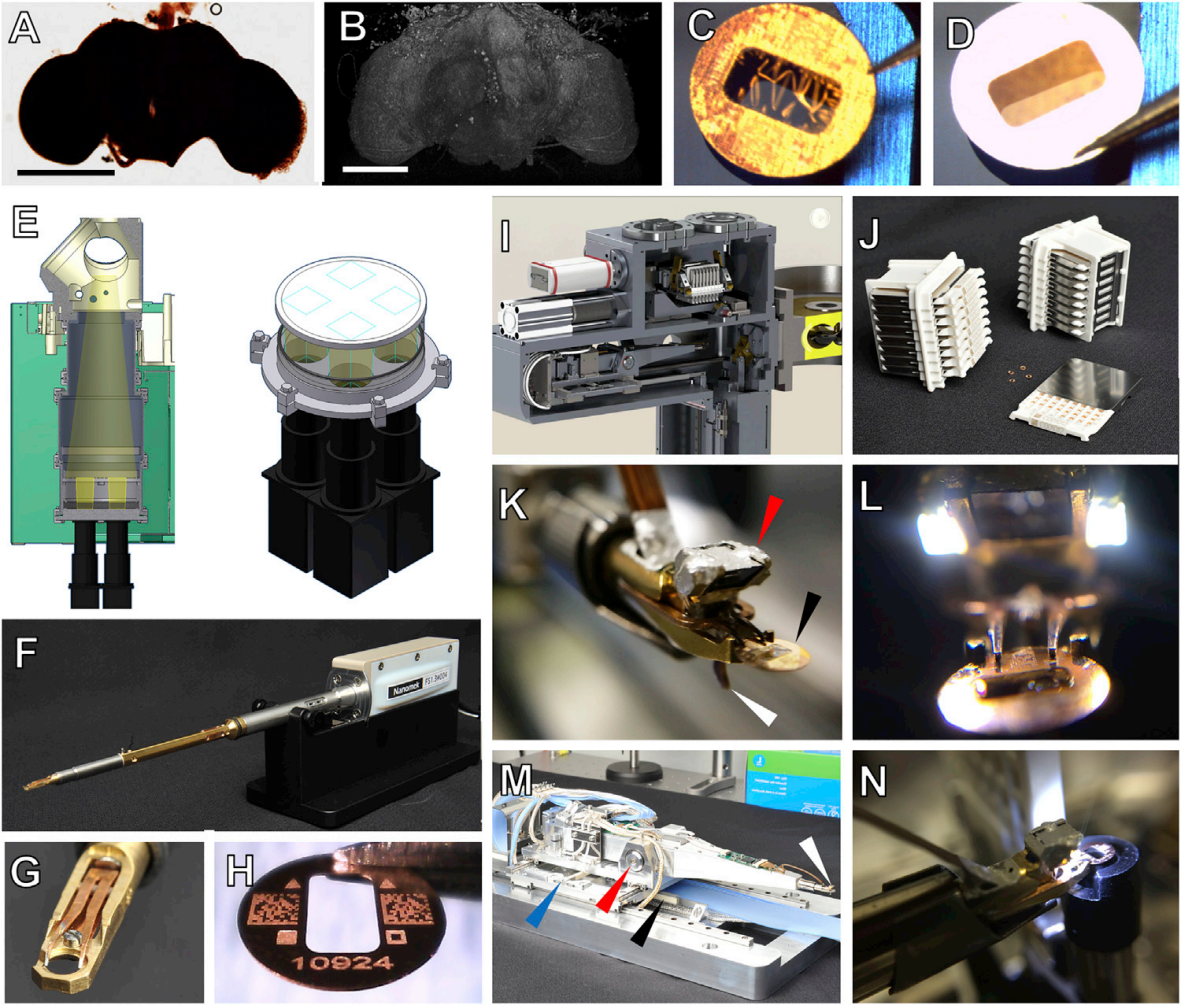
Sample Preparation and EM Acquisition Infrastructure, Related to Figure 1 (A) *Drosophila* brain following *en bloc* staining. (B) Frontal view of a 3D volumetric rendering of an X-ray tomogram from the embedded *Drosophila* brain. (C) Sample support film test showing a failed result with wrinkling of the support film on a 3 mm grid with 2 × 1 mm slot. (D) Sample support film test showing a successful result with no wrinkling or relaxation of the support film. (E) Left, schematic of TEMCA2 vacuum extension, scintillator, and camera array. Right, camera diagram showing the non-overlapping FOV of each camera on the scintillator. (F) An FEI CompuStage-compatible single-axis Fast Stage. (G) A Fast Stage grid holder. (H) A 3 mm grid with a 2 × 1 mm slot, custom-etched fiducial marks, 2D barcodes, and unique serial identifier. (I) Cassettes, magazine, and four-axis stage inside the ATPS vacuum. (J) ATPS cassettes and magazines. (K) Grid holder and end-effector of the ATPS grid positioning system. Arrows: prism and LED assembly (red); sample grid (black); lever of the grip assembly (white) which actuates grid release. (L) ATPS end-effector with LED lights for machine vision-guided pick-and-place. (M) Four-axis ATPS stage. Arrows: transverse precision piezo-driven axis (blue); pitch-axis pivot point (red); grid positioning system shuttle piezo motor (black); end effector and vision-system camera (white). (N) Rotational aligner integrated into the ATPS cassette shuttle orients the grids for imaging. Scale Bars: 250 μm in (A) and (B).

To meet the challenge of acquiring a whole fly brain, we used a variation of classical serial section TEM (ssTEM), in which images are acquired at high-speed with a TEM camera array (TEMCA) (Bock et al., 2011). Although sample handling for TEM is challenging (Figures S3A–S3D), in comparison to scanning EM-based methods, the intrinsically parallel nature of the electron optical image formed in TEM makes it relatively straightforward to achieve high-quality EM images at high-speed (reviewed in Briggman and Bock, 2012).

**Figure S2:**
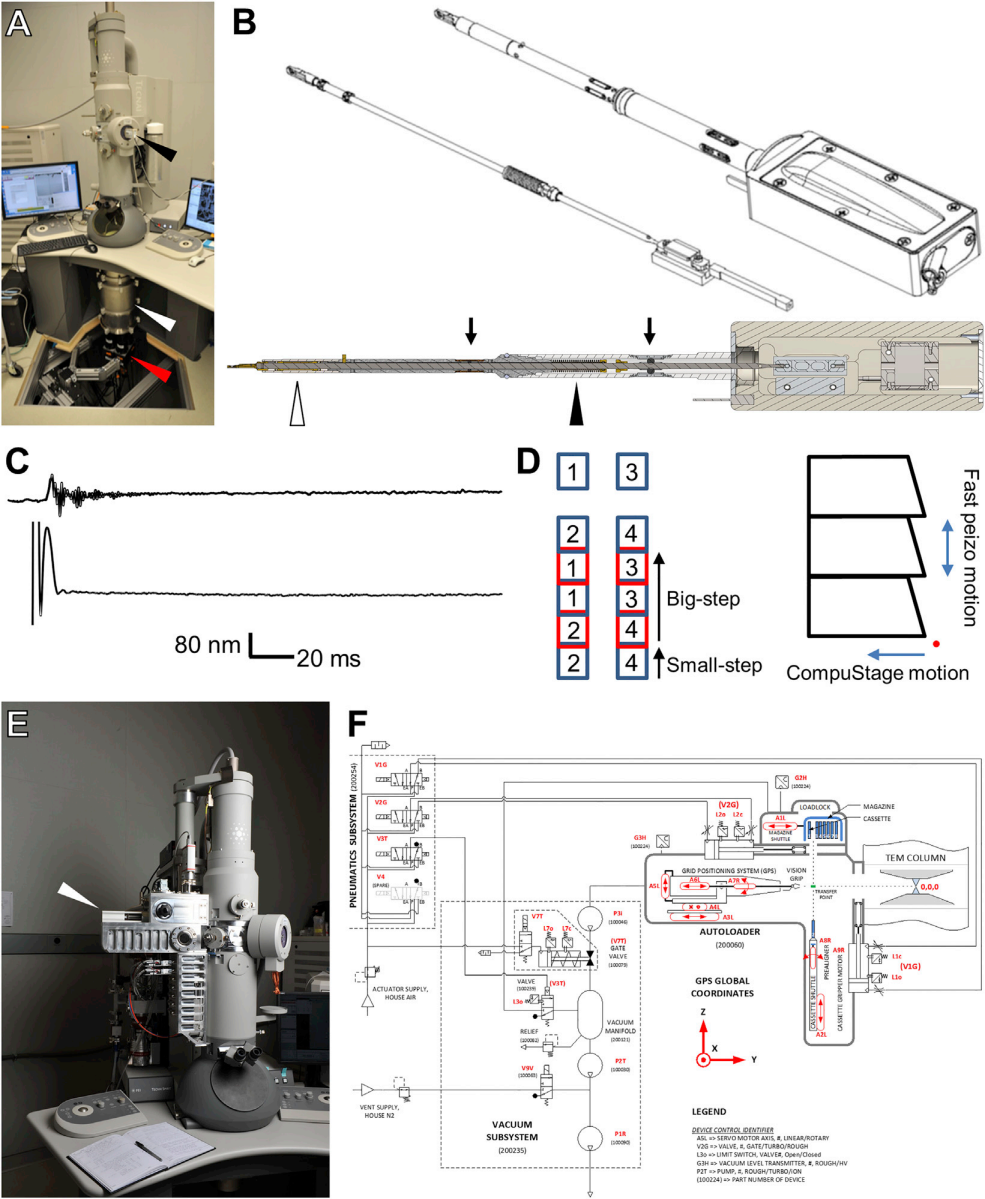
TEMCA2, Fast Stage, and ATPS for High-Throughput EM Imaging, Related to Figure 1 (A) A TEMCA2 equipped with a Fast Stage. Arrowheads: Fast Stage (black); elongated vacuum chamber (white); 2 × 2 camera array (red). (B) Fast Stage Schematic. Upper, driven mass (left) and exterior view (right). Lower, cutaway of Fast Stage showing the locations of dampers, bearings, and vacuum bellows. Arrows: rolling element damper locations (black arrows); rolling-element ‘tip’ bearing (white arrowhead); vacuum bellows (black arrowhead). (C) Plot of Fast Stage motion over time following an 8 μm move. Top trace, vibration perpendicular to Fast Stage motion axis. Bottom trace, vibration along the Fast Stage motion axis. (D) Schematic of Fast Stage stepping pattern. Left, small-step/big-step schematic. Numbers indicate camera identity within the array. Right, CompuStage and Fast Stage scanning axes. Red point is origin of scanning. (E) ATPS (white arrowhead) mounted to an accessory port on an FEI Tecnai Spirit BioTWIN TEM. (F) Schematic of the ATPS system diagraming motor positions and movement axes as well as vacuum and pneumatics subsystems.

We built two second-generation TEMCA (TEMCA2) systems (Figures S2A and S3E), using high-speed sCMOS cameras and made a custom piezo-driven Fast Stage (Figures S2B, S3F, and S3G) (Price and Bock, 2016a). Image mosaics were needed because at typical magnification, each camera in the array had an ∼8 μm field of view (FOV), whereas each whole-brain thin section was ∼750-μm wide × ∼350-μm tall (Figure 1A). At 4 nm/pixel, this resulted in a large (∼187,500 × 87,500 pixel, ∼16 GB) stitched image mosaic for each section. Each FOV was typically acquired using four 35 ms frames, versus ∼1 s in conventional TEM imaging systems. The Fast Stage moved one FOV in 30–50 ms (including settle time; Figures S2C and S2D; Video S1), versus the ∼4 s in conventional systems. The combination of high-speed imaging acquisition and sample translation allowed a single whole-brain thin section to be imaged in less than 7 min, for a *per-section* throughput of ∼50 MPix/s (∼5 times faster than the first-generation TEMCA (Bock et al., 2011), and ∼40x faster than conventional TEM systems (Takemura et al., 2013). Since each sample grid (Figure S3H) typically supported three whole-brain sections (Figure 1C), and it takes ∼10 min to exchange grids and define target regions of interest (ROIs), *per-grid* throughput, which includes sample exchange and ROI definition by microscopists, was ∼27 MPix/s.

Video S1. FEI CompuStage versus Custom Fast Stage, Related to Figure 1 The custom Fast Stage (right) makes 16 moves in the time it takes the commercial FEI CompuStage to move once. Each move is one camera FOV (∼8 μm) in length. Scale Bar: 1 μm.

To decrease overhead and to allow unattended multi-day imaging, we built a robotic Automated Transport and Positioning System (ATPS) (Figures S2E, S2F, and S3I–S3N) (Price and Bock, 2015, Price and Bock, 2016a, Price and Bock, 2016b). It enabled autonomous sample exchange in ∼5 min and autonomous imaging of TEM grids using predefined ROIs (Figure 1C; Videos S2 and S3). We mounted the ATPS on a single-camera TEM, resulting in *per-section* throughput ∼1/4 that of a TEMCA2 system. Nonetheless, the ATPS had greatly improved reliability, capacity, and throughput compared to previous TEM-based platforms for automated sample exchange and imaging (Lefman et al., 2007, Potter et al., 2004).

Video S2. ATPS Cutaway, Related to Figure 1 Computer-aided design (CAD) animation of ATPS detailing actions to retrieve grids and insert them into the TEM column.

Video S3. ATPS Pick-and-Place, Related to Figure 1 The ATPS pick-and-place routine. The ATPS locates the grid within the cassette, moves to a pre-pick location, confirms positioning, picks grid from cassette in a two-step process with positioning assessments during the process, moves to the aligner, assesses the rotational angle of the grid, if necessary places the grid on the aligner and aligns the grid, retrieves the grid from the aligner, and inserts the grid into the TEM column (insertion to column not shown). Following imaging, the ATPS locates the correct cassette pocket, confirms positioning, replaces the grid in the cassette, and confirms that the grid is correctly located within the cassette. Changes in movie quality indicate a change in camera frame rate. High quality frames (long exposure time) are used for automated calculation of grid position; lower quality frames (short exposure time) are used to generate a video log of all pick-and-place events.

### EM Image Acquisition and Volume Reconstruction of a Complete Adult Fly Brain

The above-described infrastructure provided an order-of-magnitude increase in EM imaging capacity, sufficient to image the complete brain of a female adult fruit fly at synaptic resolution in a reasonable amount of calendar time. To span the depth (∼250 μm) of the entire fly brain, 7,062 serial ∼40-nm thin sections were cut from a sample optimized for both high membrane contrast and high-quality ultrastructure (Figures 1A, 1C, and S3A). In total, 7,050 (99.8%) sections were successfully imaged, resulting in a ∼106 TB dataset comprising ∼21 million camera images.

**Figure 2:**
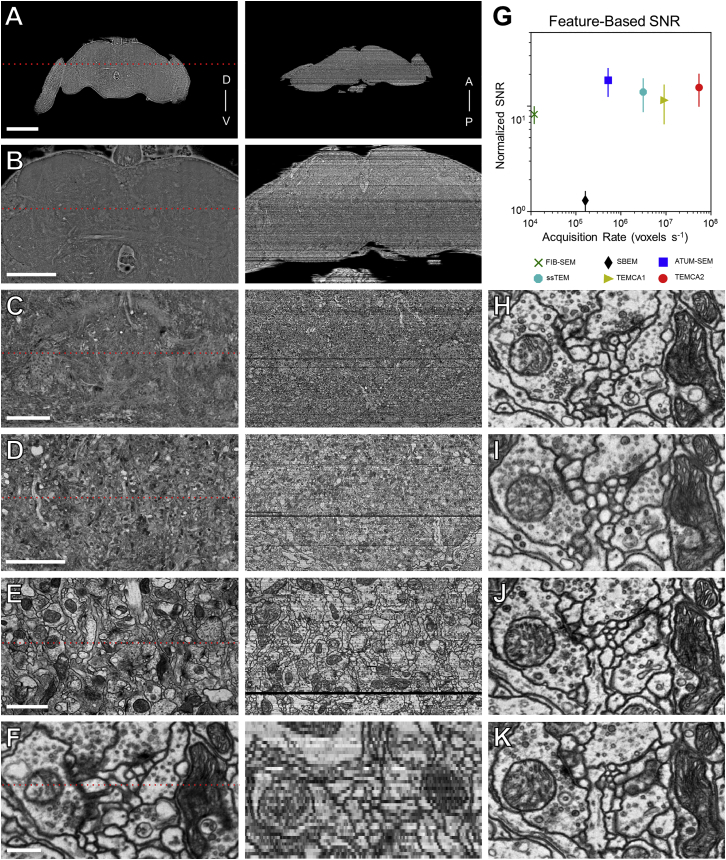
Reconstructed Image Volume (A–F) Renderings of brain-spanning EM in the sectioning plane (x-y axes) at successive zoom levels. All panels rendered using the ELM viewer (STAR Methods), which averages several adjacent sections to improve contrast at low magnifications. Red dotted lines in left column indicate orthogonal (y-z axes) section plane through the whole-brain volume, rendered in right column. (G) Image signal-to-noise ratio (SNR) versus per-section acquisition rates for the current dataset (TEMCA2) and publicly available volume EM datasets acquired via comparable techniques: FIB-scanning EM (Takemura et al., 2015), SBEM (Briggman et al., 2011), ATUM-scanning EM (Kasthuri et al., 2015), ssTEM (Takemura et al., 2013), and TEMCA (Bock et al., 2011). Error bars indicate SD. (H–K) Serial thin sections succeeding the one in (F). Fine processes can be followed across serial sections and section-to-section image registration is accurate enough to provide a consistent FOV. Axis labels are the same as those used in Figure 1. Scale bars, 200 (A), 100 (B), 25 (C), 10 (D), 2 (E), and 0.4 μm (F and H–K). See also Figures S4, S5, and S6.

**Figure S4:**
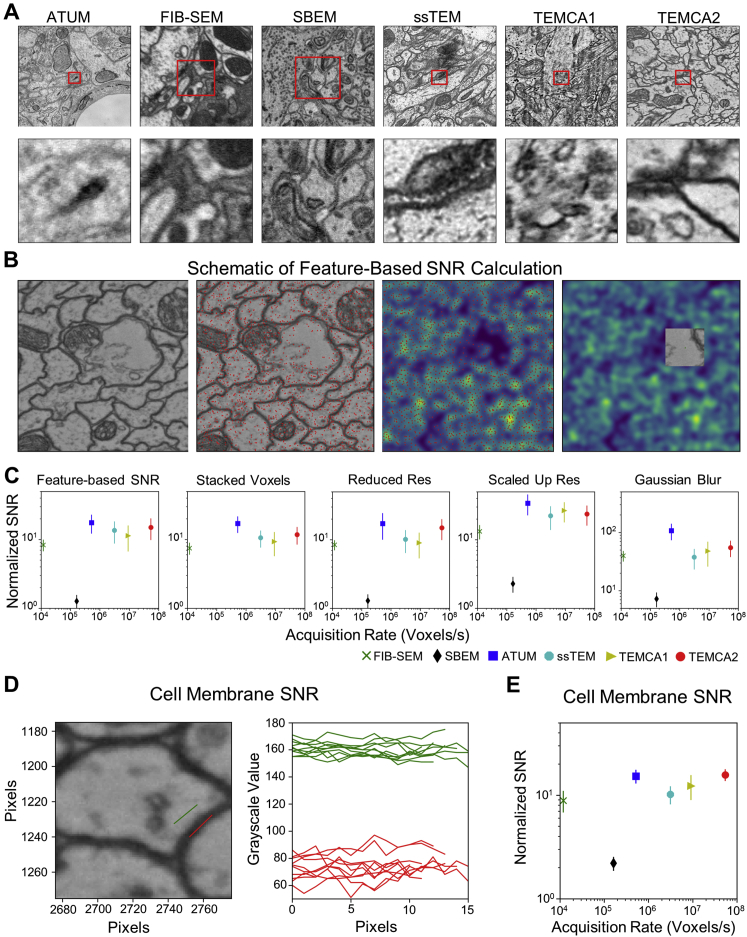
Comparison of SNR between EM Imaging Methods, Related to Figure 2 (A) Sample images from a variety of EM datasets acquired via different techniques. The data sources are as in Figure 2G. The top row shows images of side length 3 μm while the lower row shows 100 × 100 pixel subimages of each. Red squares indicate the areas of the subimages. (B) From left to right, a TEMCA2 image, the key-points detected in the image, convolution of the key-points illustrating dense and sparse feature regions (purple – low, yellow – high), the region of sparse features selected from the TEMCA2 image showing a resin filled area suitable for noise calculation. (C) The normalized SNR versus acquisition rates of a variety of EM techniques are shown for different SNR methods. Color code, points and data sources are as in Figure 2G. From left to right, the feature-based method is as described in (B); for the stacked voxels method, voxels are combined across a layer (SBEM not shown due to unclear alignments) and across 50 random images; for the reduced resolution method, voxels correspond to a larger physical size across 100 random images; for the scaled up resolution method, voxels correspond to a smaller physical size across 100 random images; for the Gaussian Blur method, voxels have been blurred with a Gaussian filter across 100 random images. Error bars indicate SD. (D) Cell membrane SNR method. Left, a representative image used to select two lines of pixels for quantifying signal (red line) and noise (green line), respectively. The pixels used for signal quantification were selected from cell membranes, and pixels used for noise quantification were selected from areas that contained only resin. Right, the grayscale values for signal (red) and noise (green) pixels selected in each region. (E) Normalized SNR versus acquisition rates as determined via the cell membrane method across five random images from each technique, each of which had 10 regions of background/noise and signal determined. Color code, points, and data sources are as in Figure 2G. Error bars indicate SD.

**Figure S5:**
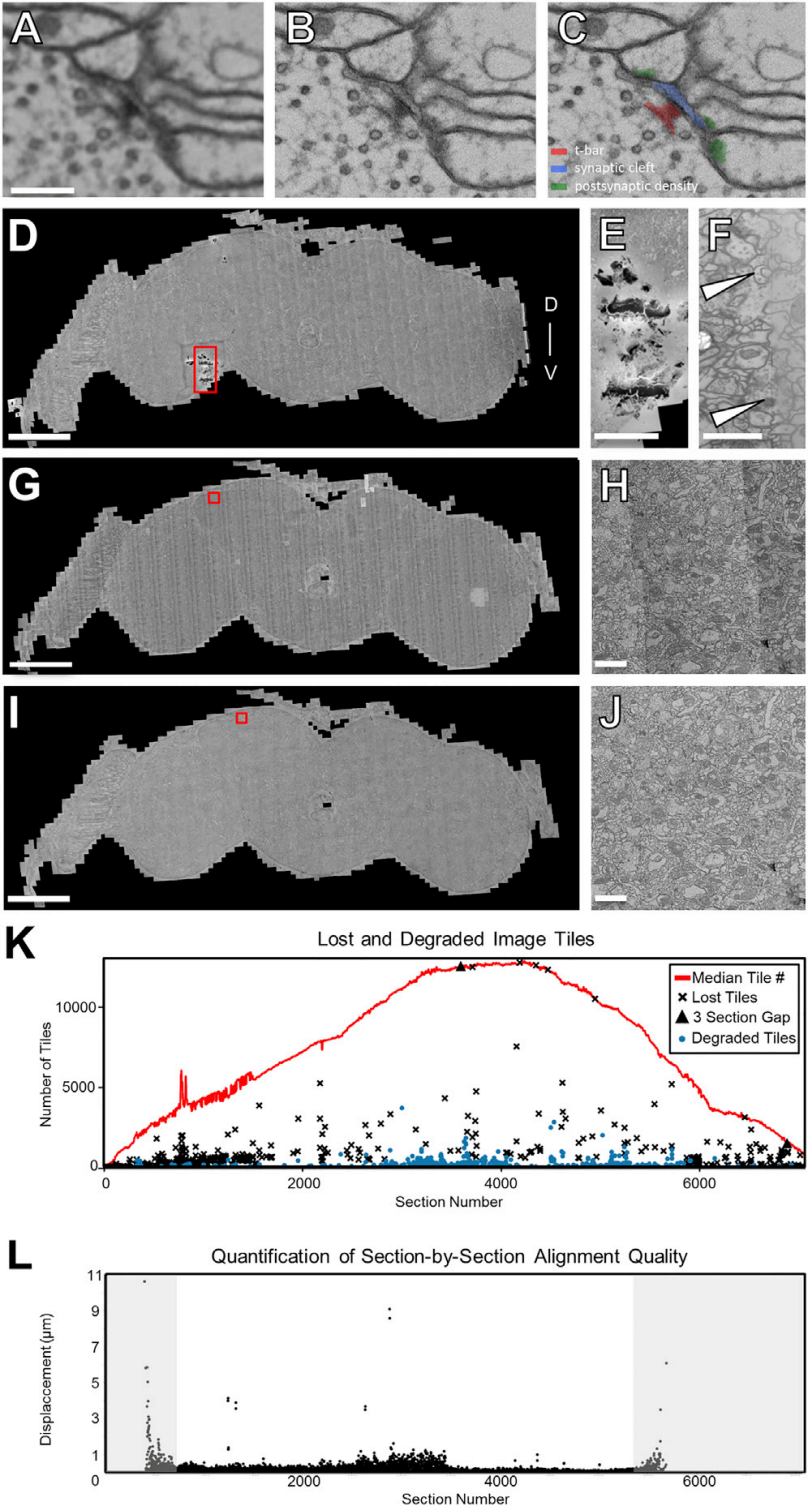
Re-imaging Synapses in MB Pedunculus, Montaging, 2D Intensity Correction, and Assessment of Volume Quality, Related to Figure 2 (A–C) Matching FOVs in section 3887 from the whole-brain volume (A) and re-imaged at higher resolution in (B-C). Resolutions in (A) and (B) and (C) are 4 nm/pixel and 0.5 nm/pixel, respectively. (D, G, and I) Whole-brain sections. (D–F) Registration of images acquired with high-dose and low-dose current beams. Debris present on a section (red rectangle) necessitated collection of a small subset of tiles at lower dose than the remainder of the mosaic. Red rectangle indicates the subregion displayed in (E). (E) A higher magnification image of the debris and border of the low-dose mosaic indicated in (D). (F) The boundary (arrowheads) in the joined high-dose and low-dose montage. (G–J) Mosaic of the same section prior to (G-H) and after (I-J) 2D intensity correction. Red squares in (G) and (I) indicate the subregions shown in (H) and (J), respectively. Intensity differences visible in (H) are greatly diminished in (J). (K) Most sections have few lost or degraded tiles. Red line, the running median of the total number of tiles per section for an 11-section window (five either side). For sections with lost tiles, only those with tile loss more than 5% below the median are shown. Triangles indicate complete loss of three consecutive sections. For sections containing degraded tiles, only those with 100 or more tiles contaminated by artifacts are shown. The dense data points and the fluctuations of running medians toward both ends of the series (sections before ∼1500 or after ∼5800) are due to tiles that contain extraneous tissue or resin outside the neuropil compartment. The tile count per section (running medians) across the series is proportional to the cross-sectional area of the brain normal to the cutting direction (z axis). (L) Volume alignment quality is sufficient for neural reconstruction. Alignment quality was assessed by analyzing the section-by-section displacements of individual Gaussian-smoothed skeletons of reconstructed neurons (STAR Methods). Large displacements are generally indicative of section misalignments. Data outside of a core region of the brain (sections < ∼500 and > ∼5500, shaded regions) are not informative since: 1) these regions mostly contain somata which typically have larger diameters than neurites, resulting in increased variability of tracing node placements, and 2) not enough tracing exists outside this range. The median and the 95% percentile of the displacements are 0.09 μm and the 0.57 μm, respectively. Scale Bars: 200 nm in (A)–(C), 100 μm in (D), (G), and (I), 25 μm in (E), 1 μm in (F), 2 μm in (H) and (J).

The quality of acquired image data was high (Figures 2, S4, and S5; Video S4). In general, image quality improves with increases to both image resolution and signal-to-noise ratio (SNR). Whether a given EM volume has sufficient resolution to reliably detect synapses and trace fine neuronal processes can only be evaluated empirically. We found that the SNR of images in the whole-brain volume equals or exceeds that of other publicly available datasets (Figures 2G and S4). If greater resolution is needed in the future, targeted ROIs can be reimaged at higher magnification (Figures S5A–S5C).

**Figure S1:**
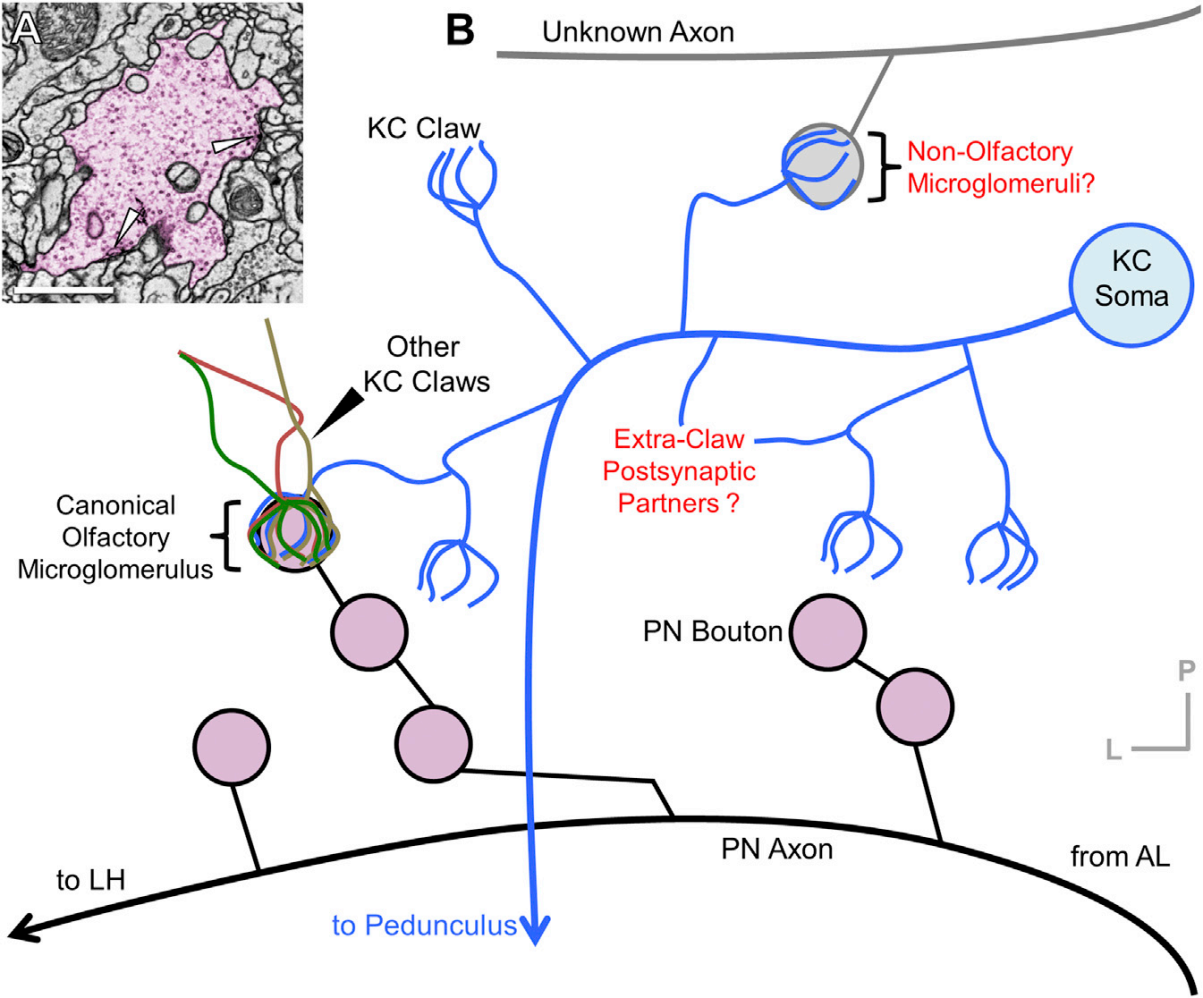
Neuronal Architecture of the MB Calyx, Related to Figure 1 (A) Micrograph of a microglomerulus in MB calyx. A canonical olfactory PN bouton (pink) is presynaptic to several fine KC dendrites, forming a synaptic complex referred to as a microglomerulus (Yasuyama et al., 2002). Arrows: presynaptic release sites. (B) Schematic of microglomerular inputs to KCs in MB calyx of *Drosophila*. The PN axons extend collaterals from the mALT into the calyx and provide bouton inputs to KCs. The *Drosophila* MB has ∼2,000 KCs on each side of the brain (Aso et al., 2014). Each KC projects a highly variable dendritic arbor into the calyx, which terminates in elaborations known as claws. Claws from many KCs wrap individual PN boutons to form each microglomerulus (Yasuyama et al., 2002), and each KC receives input from multiple PNs of diverse types across its claws (Caron et al., 2013, Gruntman and Turner, 2013). The complete composition of cell types that provide driving inputs via microglomeruli in the calyx is unknown. KCs have been shown to form presynaptic release sites in the calyx mostly outside of claws (Butcher et al., 2012, Christiansen et al., 2011), but the complete set of postsynaptic partners is unknown. Scale Bar: 1 μm in (A).

Video S4. Whole-Brain EM Volume, Related to Figure 1 All sections through the whole brain are shown in sequence. Left, a low-resolution view of each entire section. The white square is centered on the x, y position of the microglomerulus shown in Figure S1A. Right, a zoom-in of an FOV at the center of the white square. Section number 5372 shows the microglomerulus of Figure S1A.

A custom software pipeline was developed to assemble the millions of individual camera images into a coherent volume, a process known as “volume reconstruction.” A distributed compute cluster was used to stitch images from each section into a mosaic and then to register mosaics across all sections into an aligned volume (Figures 1C and 2). Artifacts and distortions introduced in serial sectioning, pickup, staining, and imaging of samples (Bock et al., 2011, Saalfeld et al., 2012), were either accommodated or corrected, resulting in a high-quality volume reconstruction of the whole brain (Figures 2 and S5; Video S4). Overall, ∼3% of camera images could not be incorporated, due to data loss during sample preparation or imaging, and ∼0.5% had poor intensity correction due to folds or EM-dense precipitates (Figures S5D, S5E, and S5K). Fewer sections were lost than in the complete *Drosophila* larval central nervous system volume, a dataset that has supported multiple significant circuit mapping efforts (Jovanic et al., 2016, Ohyama et al., 2015, Schlegel et al., 2016, Schneider-Mizell et al., 2016).

### Independent Tracing of Fine Dendrites Shows Reproducibility of Reconstruction

**Figure S6:**
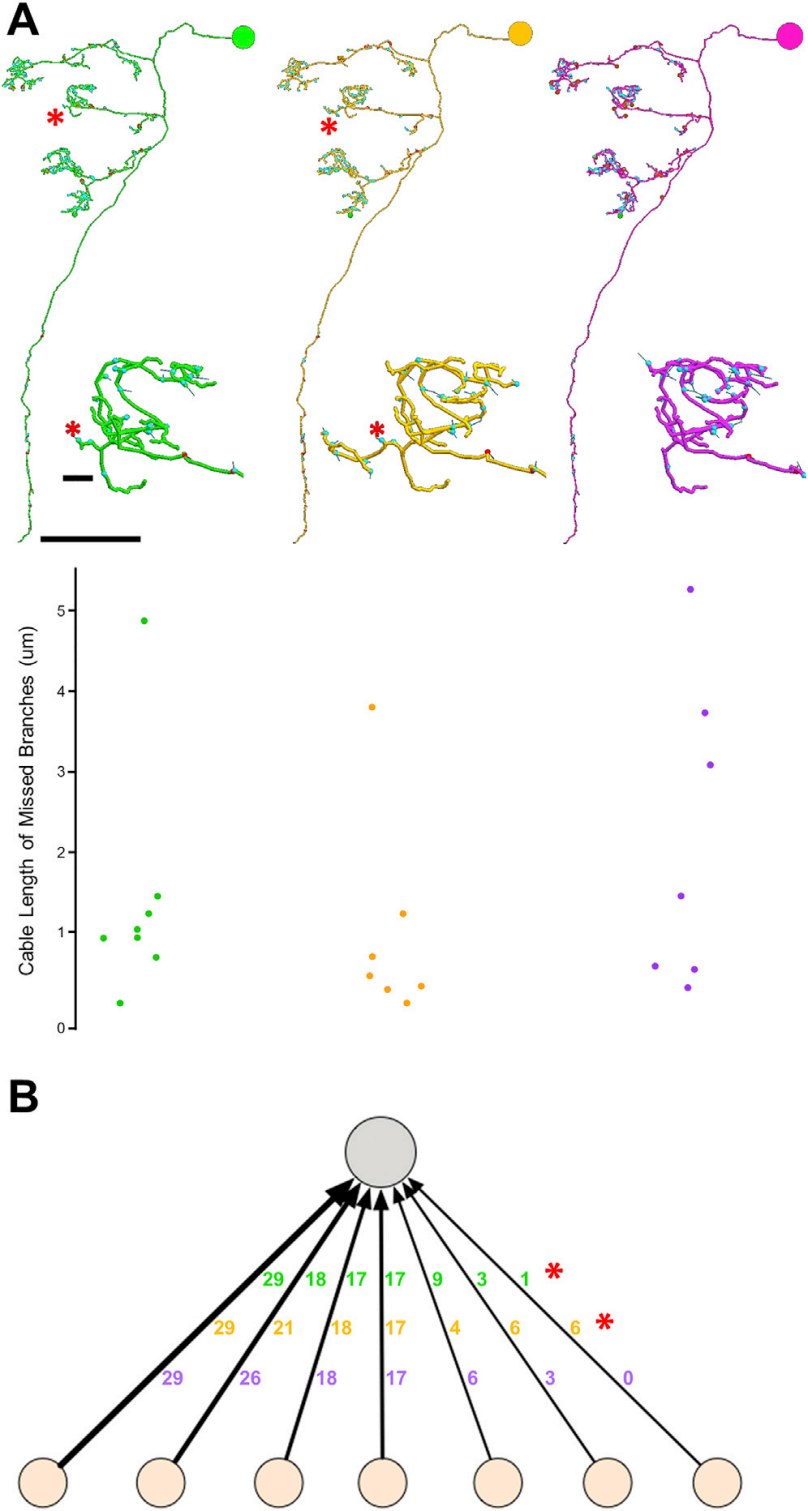
Reproducibility of Tracing, Related to Figure 2 (A and B) Three teams (indicated by colors), each comprising one tracer and one proofreader, reconstructed the same KC, with each team blinded to the others. (A) Morphologies are comparable across teams. Upper panel: asterisks indicate locations of a KC claw neurite postsynaptic to a PN input discovered by two out of three teams. Zoom-ins show the discrepancy of the fine claw neurites. Lower panel: cable length of missed branches for each of the three teams compared to an expert-generated gold-standard skeleton (STAR Methods). Consistent with Schneider-Mizell et al. (2016), the vast majority of our missed branches have a cable length of fewer than 5 μm. The reconstructed KC dendrite spanned serial sections 3451-4899. This range included one 3-section loss (3595-3597), and four single-section losses (3715, 4192, 4353, 4474), demonstrating the robustness of reproducible traceability to occasional data loss. (B) Synaptic annotations are comparable across teams. The KC (gray circle) receives input (arrows) from seven PN boutons (pale orange circles). The number of input synapses is indicated for each bouton, with the same team colors as in (A). All PN bouton inputs, except the rightmost one, were recapitulated by the three teams. Red asterisks mark the discrepant inputs caused by the missed branches in (A). Scale bars: ∼20 μm in (A), 250 nm in (A) inset.

Three teams independently reconstructed the dendritic arbor and proximal axon of a KC to completion (STAR Methods). This task was representative of challenging, fine-scale reconstruction targets, since KC dendritic claws, which ensheath PN boutons in a characteristic structure called the microglomerulus, are known to have some of the finest neurites (∼40 nm) in the fly brain (Yasuyama et al., 2002) (Figure S1A). Our tracing method extended the efficient “iterative” approach of Schneider-Mizell et al. (2016). Every neuron was initially traced and then proofread by a different team member. The dendrite claw morphologies and synaptic connections were highly consistent between the three independent reconstructions (Figure S6), and were qualitatively similar to previous reconstructions of KC dendrites in the MB calyx (Butcher et al., 2012, Yasuyama et al., 2002). In the fly brain, a useful dichotomy can be drawn between larger, microtubule-containing neurites, termed “backbones,” and fine, microtubule-free neurites that extend over short distances, termed “twigs” (Schneider-Mizell et al., 2016). In the independently reconstructed KC, backbones contained no errors, and the few discrepancies in twigs were due only to errors of omission, not commission. Our tracing approach (STAR Methods) is biased to produce this category of error, which has been shown to have minimal impact on network connectivity maps in fly (Schneider-Mizell et al., 2016). The error rate was 60.8 μm/error (454 μm total cable length; 37.9 μm/error in twigs alone; 287 μm cable length). This rate is comparable to that reported for *Drosophila* larva (27.2 μm/error, across 6 neurons totaling 2,666 μm in cable length; 16.2 μm/error in twigs alone, 1,539 μm cable length), and in mouse retina (83.4 μm/error across 1 neuron totaling 600 μm cable length; Helmstaedter et al., 2011, Schneider-Mizell et al., 2016, respectively). Thus, the independent KC reconstructions demonstrate that the EM data support accurate tracing of challenging, fine-scale neuronal morphology and connectivity.

### A Complete Survey of KC Inputs Reveals Tight Clustering of Homotypic PN Arbors

To validate that brain-spanning neuronal connectivity can be retrogradely reconstructed, the neurons providing input to all KC claw microglomeruli in the MB calyx on the right side of the brain were sufficiently traced to identify cell type. The mean reconstruction rate for all identified PNs (including tracing and proofreading of skeletons and synapses) was 120.1 μm/hr (11.2 hr per cell), and 70.1 μm/hr (11.1 hr per cell) for 15 KCs. These rates compare favorably to the ∼73 μm/h for proofread skeletons (including synapses) reported by Schneider-Mizell et al. (2016) and are comparable to 169.5 μm/hr (excluding synapses) in mammalian retina (Helmstaedter et al., 2011).

**Figure 3:**
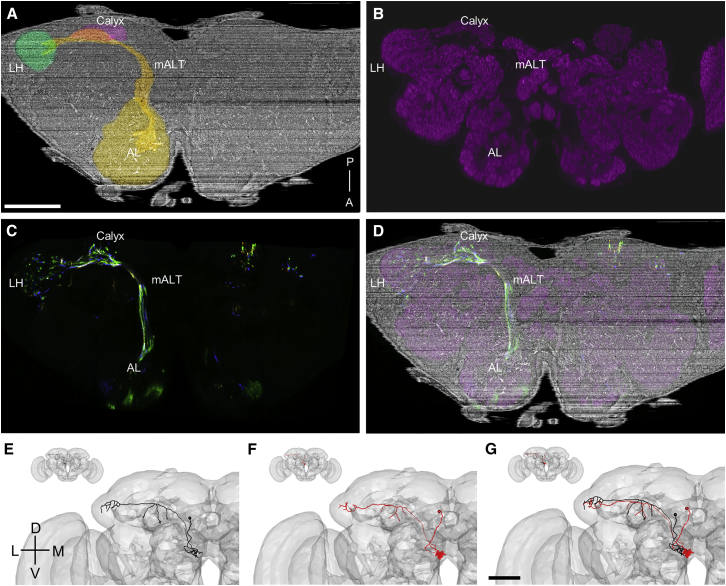
Validation of Tracing by EM-LM Registration and NBLAST-Based Geometry Matching (A) An oblique cut plane through the EM volume, selected to reveal the projection from the AL to the MB calyx and LH via the mALT. The AL, mALT, MB calyx, and LH are false colored to show compartment boundaries. (B) The LM template brain is labeled with nc82 (magenta), a synapse-specific antibody commonly used to reveal neuropil compartment boundaries (Wagh et al., 2006). After alignment to the EM volume, the same cut plane reveals corresponding neuropil compartments in both (A) and here. Nc82 labeling is absent in the mALT, a largely synapse-free PN projection tract. (C) A subset of LM-imaged PNs labeled with random fluorophore combinations (Y. Aso, personal communication) using MultiColor FlpOut (Nern et al., 2015) were registered to the template brain, and the transformation defined in (B) was used to project the PNs into the coordinate space of the EM volume. The cut plane used in (A) reveals the PN dendrites in the AL, their axonal projections in the mALT, and their axonal arborizations in the MB calyx and LH. (D) Overlaid data from the EM dataset (A), the template brain (B), and the LM-imaged PNs (C) show good co-registration between the respective whole-brain image volumes. (E) An EM-reconstructed VM2 PN (black) is projected to a template brain (gray surface mesh) using the inverse of the transformation previously defined in (B) to align the LM template brain to the EM dataset. (F) An NBLAST search of the FlyCircuit database for matches to the EM-reconstructed VM2 PN (black) returned an LM-reconstructed VM2 PN (red) as the top hit. (G) An overlay of the EM- and LM-reconstructed VM2 PNs demonstrates high qualitative similarity. Axis labels as in Figure 1. Scale bars, ∼100 (A–D) and ∼50 μm (E–G).

As expected (Caron et al., 2013, Yagi et al., 2016), most KC inputs turned out to be olfactory PNs, which are divided into genetically identified subtypes. Each PN subtype has a stereotyped and well-described morphology that spans the fly brain, and which is reproducible across individuals with a precision of ∼5 μm (Costa et al., 2016, Jefferis et al., 2007, Lin et al., 2007), allowing for quantitative assessment of the EM-based reconstructions. Morphological reconstructions of LM-imaged PNs from most subtypes are available in online databases, and have been aligned to a small number of LM-imaged template brains (Chiang et al., 2011, Manton et al., 2014, Milyaev et al., 2012). Alignment of an LM-imaged template brain (Aso et al., 2014) to the EM volume (STAR Methods) allowed LM-imaged neurons from a volume previously aligned to the template brain to be overlaid on the EM dataset (Figures 3A–3D).

Quantitative comparisons of EM- and LM-reconstructed PNs were made using NBLAST (Costa et al., 2016), which measures similarity between neuronal arbors. Similarity scores between EM- and LM-reconstructed PNs of the same subtype were high. In nearly all cases, the top NBLAST hit agreed with expert identification of PN subtype (Table S1). EM-to-LM similarity scores were comparable to LM-to-LM scores. For example, the six VM2 PNs in an LM morphological database (FlyCircuit) (Chiang et al., 2011), when compared with one another, resulted in NBLAST scores of 0.64–0.71 (scale of −1 to 1, where 1 indicates identical neuron shape and position), and the EM-reconstructed VM2 PN returned an NBLAST score of 0.64 when compared to the same PNs. Qualitative assessment of similarity agreed with the quantitative scoring (Figures 3E–3G). Since the LM-imaged template brains have also been aligned to one another (Manton et al., 2014), this tool chain can be used to quantitatively compare any aligned LM- and EM-reconstructed neurons.

**Figure 4:**
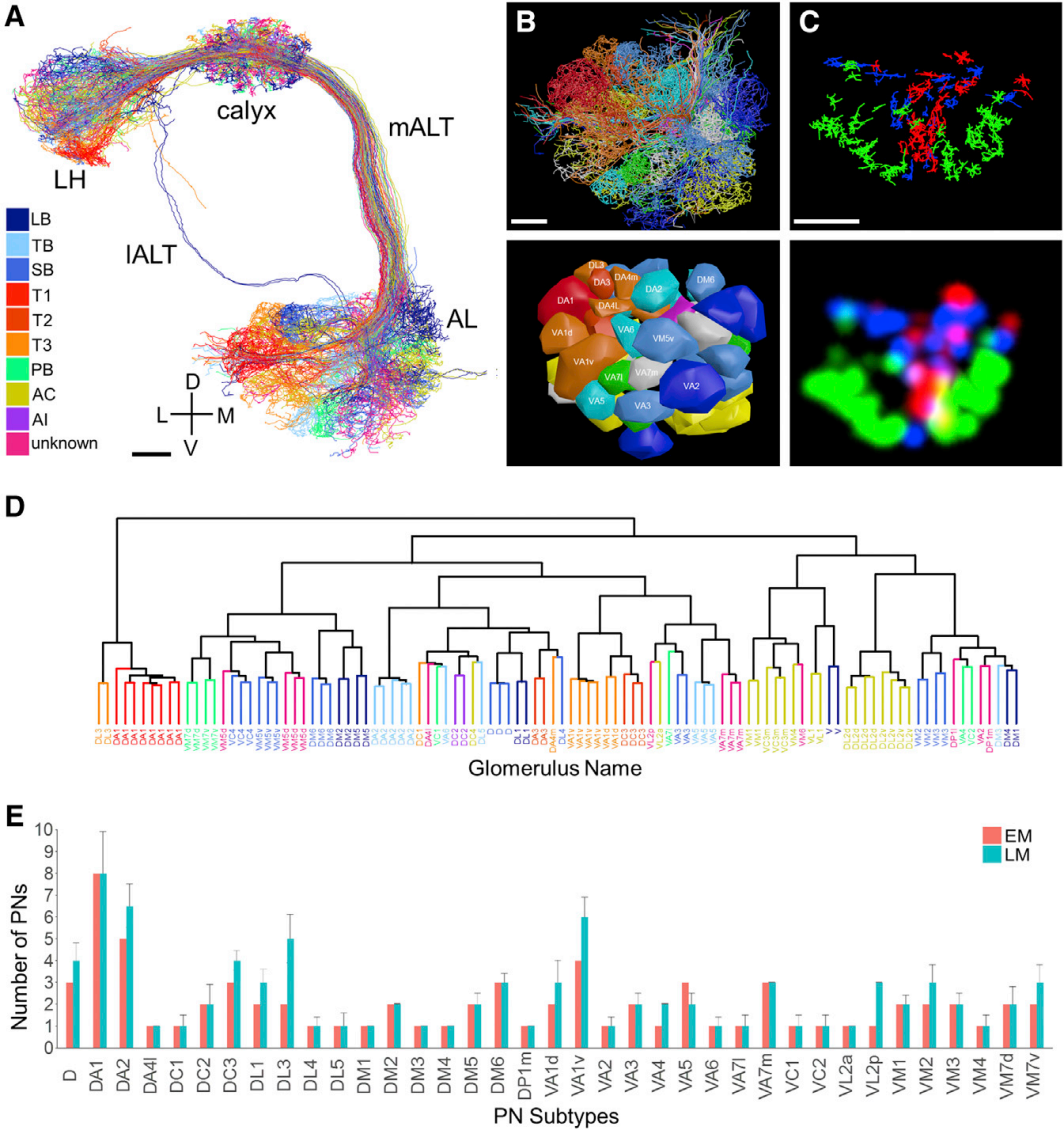
Survey of Olfactory PNs Providing Driving Input to Microglomeruli in the MB Calyx Agrees with LM Data (A) EM-reconstructed uniglomerular olfactory PNs in the right hemisphere recapitulate known olfactory pathways (summarized in Figure 1B). (B) A frontal view of EM-reconstructed PNs (top) and glomerular surface models (bottom) in AL shows agreement with previous glomeruli reconstructions (Couto et al., 2005, Grabe et al., 2015). (C) A frontal-dorsal view of EM-reconstructed boutons for three groups of PNs in MB calyx reveals concentric organization, consistent with LM data (Tanaka et al., 2004). Bouton skeletons (top) were used to generate Gaussian-smoothed bouton volumes (bottom; STAR Methods) for each of the three groups. The PN groups are DM1, VA4, VC1, and VM2 (green); DL1 and VA6 (blue); and DA1, DC3, and VA1d (red). (D) Unsupervised clustering based on morphological similarity (NBLAST score) produces a dendrogram in which olfactory PNs are grouped by glomerular subtype. (E) Comparison of the number of reconstructed PNs per glomerulus from EM and LM data (Grabe et al., 2016). Colors in (A), (B), and (D) like in Couto et al. (2005). PNs receive input from olfactory receptor neurons (ORNs). The dendrite of each ORN innervates an antennal protuberance called a sensillum. Each PN is colored by the class of sensillum its input ORNs innervate. Error bars indicate SD. Axis and anatomical labels are the same as those used in Figure 1; lALT, lateral antennal lobe tract; LB, large basiconic; TB, thin basiconic; SB, small basiconic; T1, T2, T3, trichoid sensilla; PB, maxillary palp basiconic; AC, antennal coeloconic; AI, antennal intermediate. Scale bars, ∼10 μm (A–C). See also Table S1.

Of the 578 boutons providing input to KC claws, 497 arose from olfactory PNs (86%, from 114 PNs). The PN dendrites in the antennal lobe (AL) permitted identification of all 50 known olfactory glomeruli (Grabe et al., 2015) (Figures 4A–4C), although two could not be disambiguated (VC5 and VC3l; STAR Methods). All classified PNs are included in a downloadable tracing environment for use in future efforts mapping olfactory circuits in the fly brain (Key Resources Table; STAR Methods). Seventeen boutons (3%) arose from a previously unknown neuron that we named “MB-CP2” (“Mushroom Body Calyx Pedunculus #2,” per the naming convention of Tanaka et al. (2008) and further describe below. The remaining 11% of boutons come from other unidentified (presumably non-olfactory) PN types and other cell types from outside the AL. Consistent with previous LM data, the arbors of specific PN subtypes formed concentric clusters in MB calyx (Figure 4C; compare to Figure 4 in Tanaka et al., 2004). Unsupervised clustering based on NBLAST scores grouped homotypic PNs (Figure 4D). The number of PNs arising from each glomerulus (Figure 4E) was consistent with recent LM data (Grabe et al., 2016).

**Figure 5:**
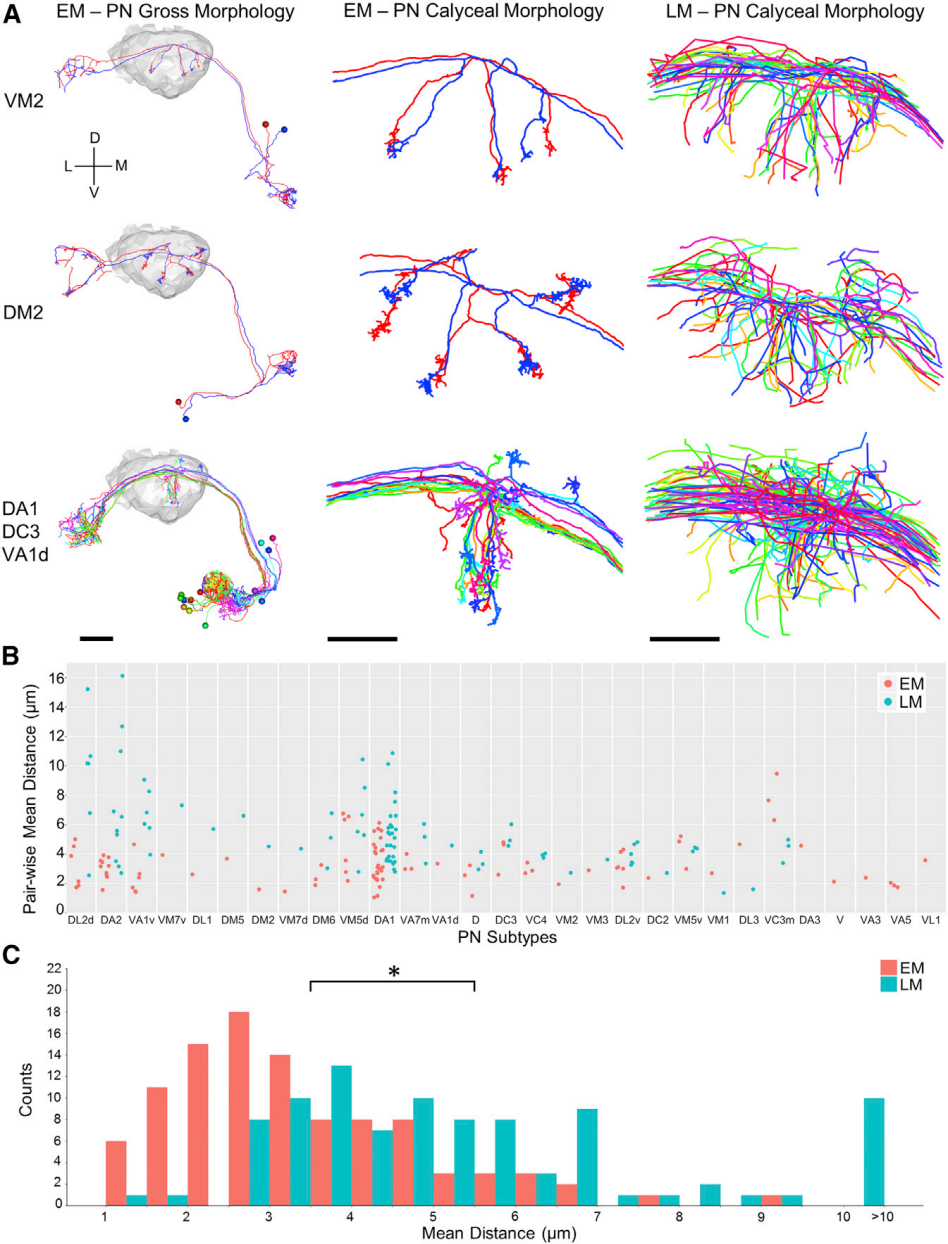
PN Arbors in MB Calyx Cluster More Tightly than Previously Seen with LM across Individuals (A) Comparison of EM- versus LM-reconstructed PNs. EM-reconstructed PNs are shown against a surface model of MB calyx (gray) in the left column. Calyx arbors for EM- and LM-reconstructed PNs are shown in the middle and right columns, respectively. Data for LM-reconstructed PNs (right column) are from the FlyCircuit database (Chiang et al., 2011), as registered to a common template brain (Costa et al., 2016; see also STAR Methods). (B) Pairwise distances between homotypic PN collaterals in the MB calyx. Each data point represents the distance between one pair of EM- (red) or LM-reconstructed (blue) PNs from the same subtype. Data points are bucketed according to PN subtype; subtypes are ordered on the x axis by how much more clustered EM-reconstructed PNs are than LM-reconstructed PNs (STAR Methods). (C) Histogram of all data points in (B). The mean of pairwise distances for all EM-reconstructed PN subtypes was significantly lower than that for all LM-reconstructed PN subtypes (3.40 ± 1.53 μm vs. 5.49 ± 2.73 μm, respectively; Student’s t test, p < 1.3 × 10^−9^). Axis labels as in Figure 1. Scale bars, ∼20 (A, left column) and ∼10 μm (A, middle and right columns). See also Figure S7.

**Figure S7:**
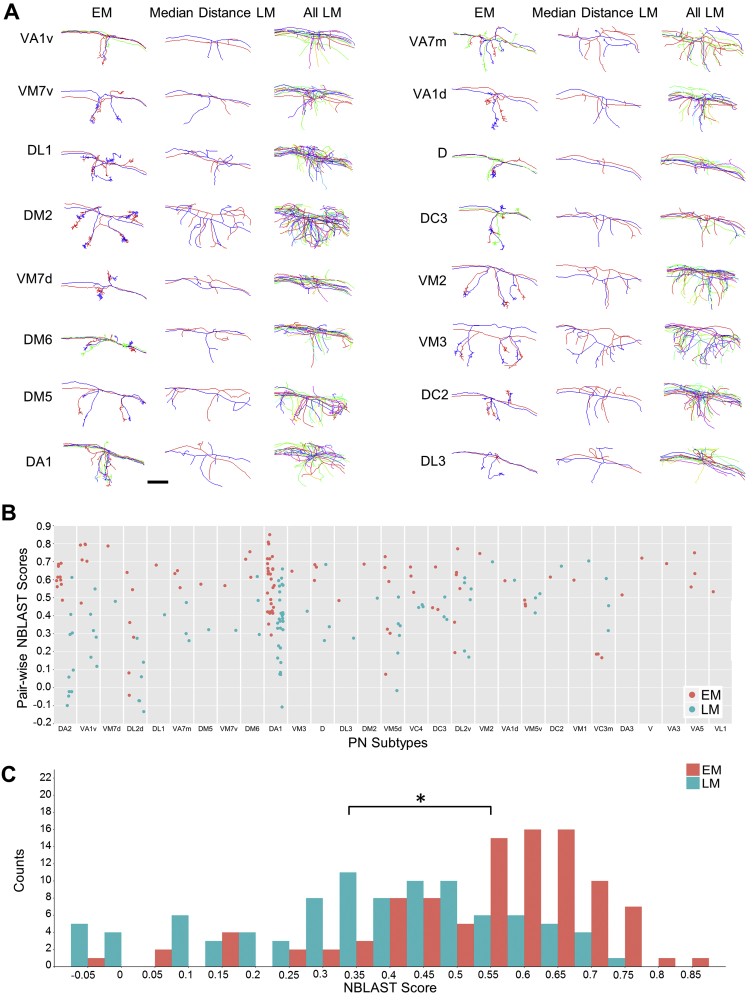
Comparison of MB Calyx Collaterals Reveals Greater Similarity between EM-Reconstructed PNs than LM-Reconstructed PNs, Related to Figure 5 (A) Pairs of EM-reconstructed PNs are qualitatively more similar than pairs of LM-reconstructed PNs in the MB calyx. Subtypes are ordered by how much more clustered EM-reconstructed PNs are than LM-reconstructed PNs (STAR Methods). Middle column shows the pair of LM-reconstructed PNs with the median pairwise distance across all pairs. (B) Pairwise NBLAST scores between homotypic PN collaterals in the MB calyx. Each data point represents the NBLAST scores between one pair of EM- (red) or LM-reconstructed (blue) PNs. Data points are bucketed according to PN subtype; subtypes are ordered on the x axis by how much more similar EM-reconstructed PNs are than LM-reconstructed PNs (STAR Methods). (C) Histogram of all data points in (B). The mean of pairwise NBLAST scores for all EM-reconstructed PN subtypes was significantly higher than that for all LM-reconstructed PN subtypes (0.56 ± 0.18 versus 0.35 ± 0.21, respectively; Student’s t test, p < 2.2 × 10^−12^), indicating that EM-reconstructed PN subtypes are morphologically more similar to each other than LM-reconstructed PN subtypes. Scale bar: ∼10 μm in (A).

Previously, LM data pooled across multiple animals showed that homotypic PN collaterals fasciculate tightly in lateral horn, but only loosely (or not at all) in MB calyx (Jefferis et al., 2007). In contrast, our reconstructions in an individual brain showed that in MB calyx, homotypic PNs often cluster tightly (Figures 5 and S7). The PN cluster at the center of the concentrically arranged arbors shown in Figure 4C was also tighter in the EM data than in LM data pooled across multiple animals (Figure 5A, bottom row). Quantification of the average distance between homotypic PNs revealed that intra-animal arbors are significantly more clustered than arbors from multi-animal LM data (Student’s t test, p < 1.3 × 10^−9^) (Figures 5B and 5C). A similar result was obtained based on NBLAST score differences (Student’s t test, p < 2.2 × 10^−12^) (Figures S7B and S7C). The tight clustering of EM-traced PNs suggests developmental co-fasciculation of homotypic inputs to the MB calyx, and may bias the KC sampling of olfactory input, in contrast to a purely random model based on data pooled across multiple individuals (Caron et al., 2013; see the Discussion). These results indicate that the whole-brain EM dataset supports accurate and efficient tracing of PNs, a representative brain-spanning cell type.

### A Previously Unknown Cell Type, MB-CP2, Provides Input to KC Claws

**Figure 6:**
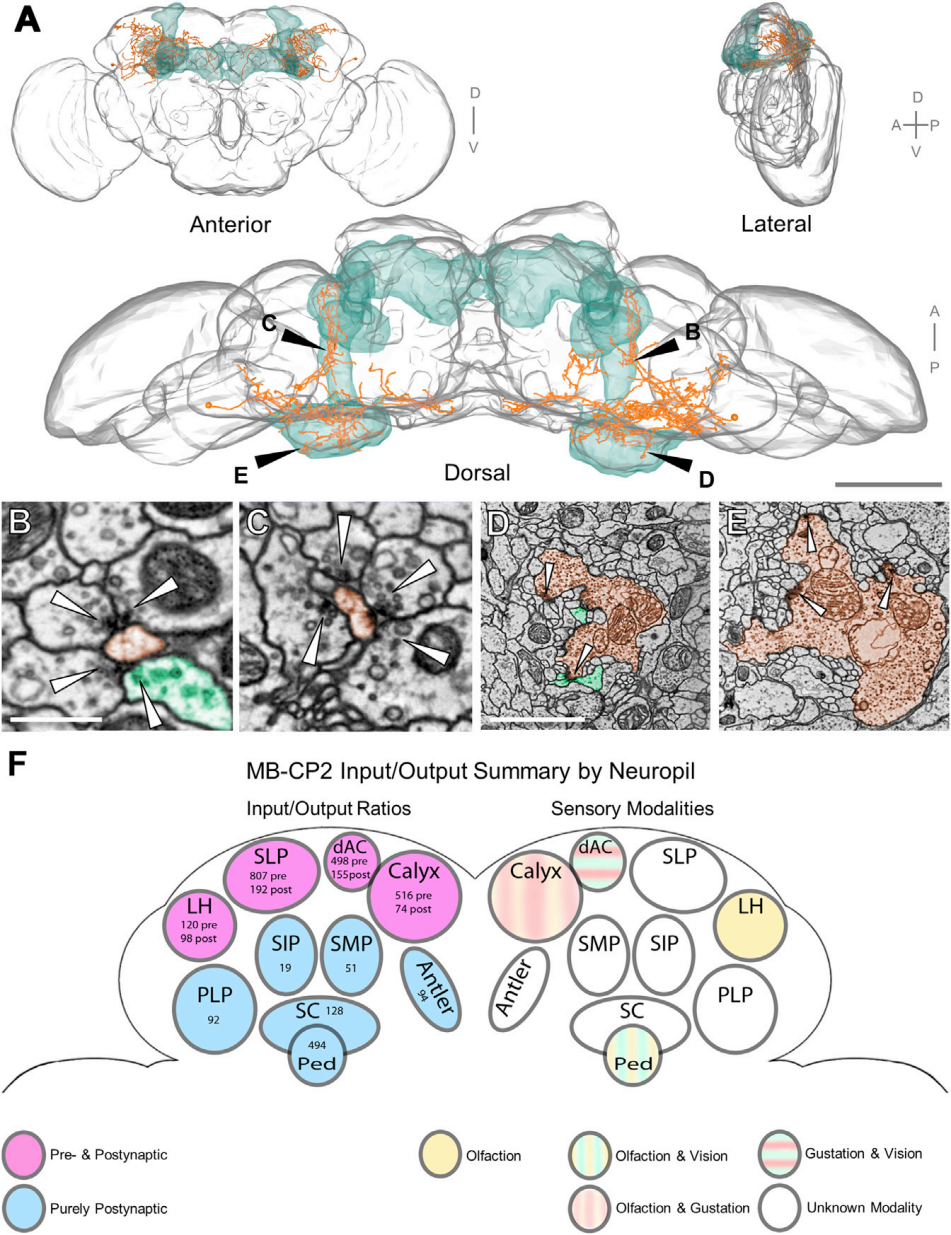
MB-CP2, a New Cell Type Providing Microglomerular Input to KC Claws (A) Reconstruction of the pair of MB-CP2 neurons with surface meshes of the whole brain (gray) and MB (green). (B–E) Synaptic connectivity between MB-CP2 and KCs in MB pedunculus and main calyx. Since many KC claws ensheath a given MB-CP2 bouton and KCs are traced only sparsely in this study, most postsynaptic profiles are untraced (STAR Methods). Arrowheads, presynaptic release sites. (B) A representative MB-CP2 neurite (orange) in MB pedunculus, postsynaptic to a KC axon (green). (C) A representative MB-CP2 neurite (orange) in left hemisphere MB pedunculus, with comparable synaptic arrangements to (B). KCs were not traced in the left hemisphere, so cell identity is putative. However, most bundled neurites parallel to the MB pedunculus long axis arise from KCs (Leitch and Laurent, 1996, Schürmann, 2016; data not shown). (D) A cross-section through a representative MB-CP2 bouton (orange) in MB main calyx at the center of a canonical microglomerulus. Several postsynaptic profiles arise from KC claws (green). (E) A representative MB-CP2 bouton in left hemisphere MB main calyx, with comparable synaptic arrangements to (D). KCs were not traced in left hemisphere, so cell identity is putative; however, most postsynaptic elements at MB calyx microglomeruli arise from KCs (Butcher et al., 2012; data not shown). (F) Summary schematic of MB-CP2 input and output brain regions with synapse counts discovered following partial reconstruction (STAR Methods). In six brain regions, MB-CP2 dendrites are purely postsynaptic. In four other regions, MB-CP2 neurites are both pre- and postsynaptic. Axis and anatomical labels as in Figure 1; Ped, MB pedunculus; dAC, dorsal accessory calyx; ATL, antler; SC, superior clamp; PLP, posterior lateral protocerebrum; SMP, superior medial protocerebrum; SIP, superior intermediate protocerebrum; SLP, superior lateral protocerebrum. Scale bars, 100 μm (A, dorsal view), 500 nm (B and C), and 2 μm (D and E). See also Video S5.

To better characterize MB-CP2, we traced the entire backbone of MB-CP2, which proved to be extensive throughout the ipsilateral (right) hemisphere. A second MB-CP2 neuron was located in the left hemisphere. Reconstruction of its complete backbone revealed symmetrical arborization in the equivalent contralateral neuropil compartments, which is typical of most cell types in the fly brain (Aso et al., 2014, Chiang et al., 2011, Jenett et al., 2012, Ohyama et al., 2015). This confirmed MB-CP2 as a bona fide morphological cell type (Figure 6; Video S5).

Video S5. Synapses in Neuropil Compartments Innervated by MB-CP2, Related to Figure 6 The MB-CP2 neurons (orange skeletons) are shown inside the whole-brain surface mesh (gray) and other brain region meshes (multiple colors). In the right hemisphere, MB-CP2 is purely postsynaptic (blue dots) in the MB pedunculus, ATL, LH, PLP, SCL, SMP, SIP, and SLP (synapses shown first). MB-CP2 is both presynaptic (red dots) and postsynaptic in the dAC (surface mesh not shown), LH, and SLP. In the MB, MB-CP2 is postsynaptic in the pedunculus and both pre- and postsynaptic in the calyx (synapses shown second), forming MB recurrent circuits. See Figure 6F for synaptic input/output summary schematic by brain region. Video begins from an anterior perspective with dorsal at top and ventral at bottom.

MB-CP2 boutons are presynaptic to all five known olfactory KC subtypes (γ, αβc, αβs, α′β′m, α′β′ap) in MB main calyx (Aso et al., 2014). In the dorsal accessory calyx (dAC), MB-CP2 boutons also provide input to αβp KCs (data not shown). In all brain regions where MB-CP2 makes output synapses, its neurites also receive input synapses (Figure 6F). In the MB pedunculus, MB-CP2 receives input from γ KCs (Figure 6B) and γd KCs, a subtype originating in the ventral accessory calyx known to receive visual inputs (Yagi et al., 2016; data not shown). Many areas where MB-CP2 is purely postsynaptic are innervated by multimodal sensory and motor neurons (e.g., SMP and PLP; Hsu and Bhandawat, 2016, Namiki et al., 2017). Therefore, MB-CP2 neurons likely relay multimodal, non-olfactory input to KCs, and provide recurrent feedback from KC axons in the MB pedunculus to KC dendrites in the MB main calyx, adding to the set of known recurrent circuits in the MB (Aso et al., 2014, Owald and Waddell, 2015).

### Identification of Cell Types Postsynaptic to KCs in the MB Calyx

**Figure 7:**
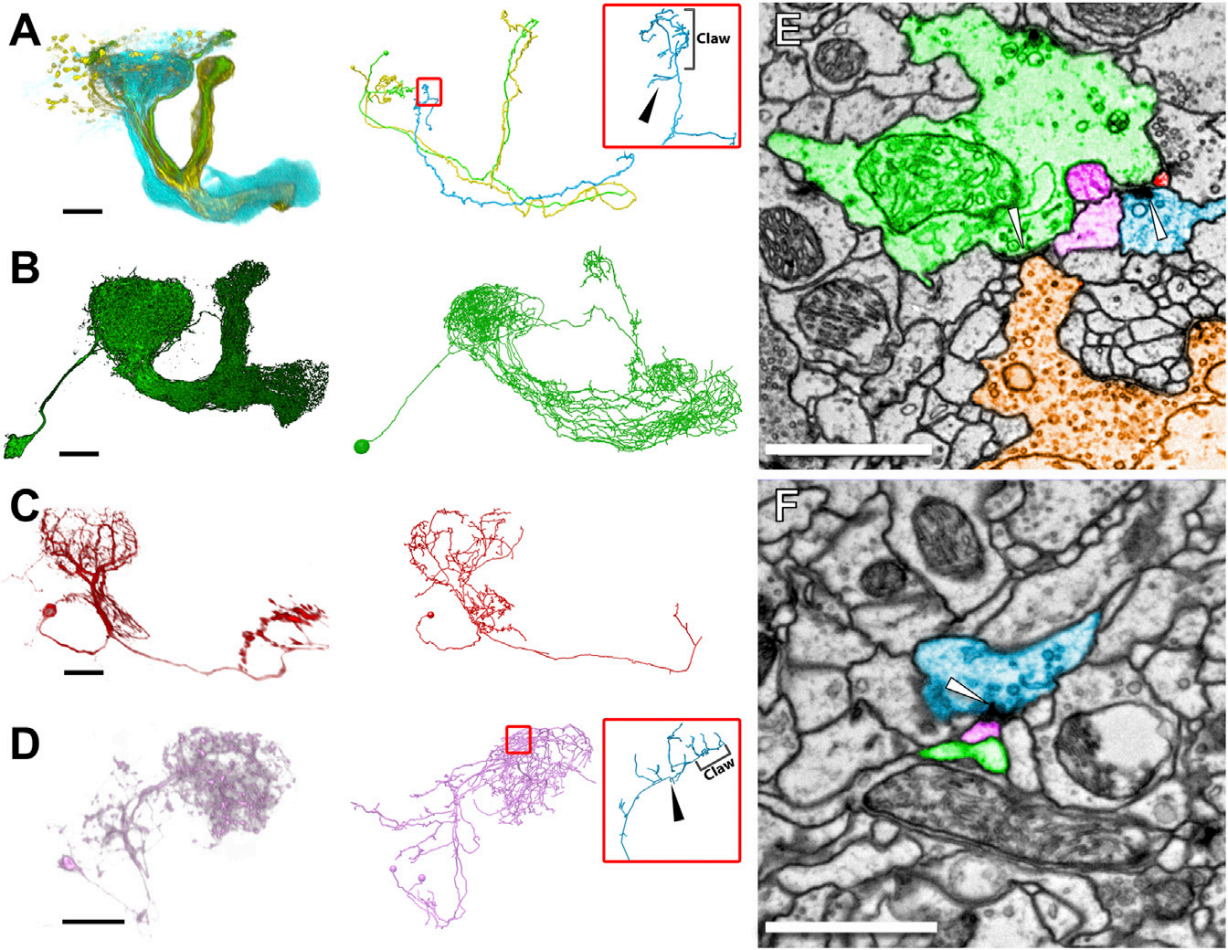
KC Presynaptic Release Sites in the MB Calyx Mostly Target a Small Subset of Available Partners (A–D) Morphological comparison of LM-imaged (left panels) and EM-reconstructed (right panels) neurons of the same class. LM data from Aso et al. (2014). Neurite densities are lower in the EM reconstructions, since these cells were traced to classification, not completion (STAR Methods). Spheres in the EM-reconstructions indicate the location of cell bodies. (A) αβc- (green), αβs- (yellow), and γ- (cyan) KCs. One representative EM-reconstructed KC from each class is shown (right panel); their morphologies and trajectories match those of the LM-imaged KCs of the same class. Small red square: location in MB calyx of a γ KC sub-arbor shown in the inset (large red square). Inset: representative location of a presynaptic release site (black arrowhead), on a twig arising from the KC backbone, outside the claw sub-arbor. A micrograph of this site is shown in (E). (B) APL, a wide-field inhibitory neuron that innervates the entire MB and sparsifies KC activity (Lin et al., 2014, Liu and Davis, 2009). (C) MB-CP1, a MB output neuron (MBON) with a dendritic arbor innervating the MB calyx and pedunculus (Tanaka et al., 2008). (D) MB-C1, a putative inhibitory interneuron that innervates the MB calyx and LH (Tanaka et al., 2008). Two MB-C1 neurons were found in the EM-based survey of KC postsynaptic targets, in contrast to the single neuron reported by Tanaka et al. (2008). Small red square: location in MB calyx of a γ KC sub-arbor shown in the inset (large red square). Inset: representative location of a presynaptic release site (black arrowhead), on a twig arising from the KC backbone, outside the claw sub-arbor. A micrograph of this site is shown in (F). For clarity, this KC is not shown in (D, right panel). (E and F) Micrographs at the synapse locations shown in (A) and (D) insets. Arrowheads, selected presynaptic release sites. (E) The γ KC in (A, inset) and two other γ KCs (light and dark purple) that are presynaptic to APL (green), MB-CP1 (red), and each other at the same synaptic cleft. The APL is also presynaptic to a PN (brown). (F) The γ KC from (D, inset) is presynaptic to MB-C1 (pink), APL (green), and several additional unidentified partners. The APL postsynaptic density is two sections away (not visible in this section plane). Scale bars, ∼25 (A–D) and 1 μm (E and F). See also Table S2.

In addition to their canonical input from PN boutons, KC dendrites make synaptic outputs to unknown targets in the MB calyx, forming a poorly understood local microcircuit (Butcher et al., 2012, Christiansen et al., 2011). To validate use of the whole-brain dataset for anterograde mapping of synaptic connectivity, we reconstructed and identified the postsynaptic targets of 15 KCs in the MB calyx. All dendritic presynaptic release sites were annotated on three KCs from each of the five subtypes in MB main calyx. Consistent with immunohistochemical data (Christiansen et al., 2011), most (82%) presynaptic release sites arise from αβc-, αβs-, or γ KCs, and 87% of all release sites are distributed along KC dendrites outside the claws (Figure 7; Table S2). Fourteen percent of fine postsynaptic neurites were too difficult to trace to a parent backbone with high confidence. All KC presynaptic release sites divergently target multiple postsynaptic processes, which in many cases arise from identified MB intrinsic and extrinsic neurons and form local recurrent microcircuits (Figures 7E and 7F).

We found that KCs preferentially target a subset of potential partner cell types in the MB calyx, with only four of the 14 available cell types (STAR Methods) making up 75% of the postsynaptic targets (Figure 7; Table S2). Intriguingly, α′β′ KCs (which are dispensable for memory retrieval) (Krashes et al., 2007) were even more selective, only synapsing onto APL and other KCs. This selectivity could arise because either the neglected cell types or α′β′ KCs arborize sparsely in the MB calyx; alternatively, they could be specifically neglected despite extensive arbor overlap and high availability of potential synapses (Stepanyants and Chklovskii, 2005). To distinguish between these alternatives, future work should sample more KCs and reconstruct to completion available postsynaptic cell types.

## Discussion

Here, we contribute a complete EM volume of an adult female *Drosophila* brain for free use by the research community. Validation reconstructions demonstrated the reliable tracing of local and long-range connectivity, revealing both known and new elements in the MB circuitry underlying associative learning and recall. We conclude this volume is suitable for tracing brain-spanning neuronal circuits at synaptic resolution. Reconstructions of the neurons reported here are included in a preconfigured, downloadable tracing environment to serve as entry points for further investigation.

Generation of the whole-brain dataset was enabled by new high-throughput hardware and scalable software for acquiring and aligning large-scale EM data. Although improvements to TEMCA throughput are readily achievable, alternative volume EM imaging approaches such as multibeam-scanning EM and FIB-scanning EM also show great promise (Eberle et al., 2015, Xu et al., 2017). In larger animals, brain-spanning connectomics may be achieved by low resolution EM imaging, followed by high resolution reimaging of synaptic connectivity in selected sub-volumes (Hildebrand et al., 2017). Although the alignment quality shown here sufficed for manual tracing, further improvements, such as better fine-scale registration near artifacts, may be needed to fully leverage emerging methods for automatic segmentation (Arganda-Carreras et al., 2017, Beier et al., 2017, Januszewski et al., 2016). Early segmentation results on subsets of this whole-brain dataset are nonetheless promising (Funke et al., 2016).

The alignment of template LM brains to the whole-brain EM volume allows EM-reconstructed neurons to be searched for in large on-line databases of morphologically stereotyped cell types. Genetic driver lines for cell types (Aso et al., 2014, Grabe et al., 2015, Jenett et al., 2012, Kvon et al., 2014) discovered to participate in a given EM-reconstructed circuit can therefore be readily identified. This will facilitate testing of physiological and behavioral hypotheses generated by newly mapped EM wiring diagrams.

Our finding that olfactory PN arbors in the MB calyx cluster much more tightly than predicted by LM data pooled across animals is consistent with observations for pairs of neurons from one glomerulus by Kazama and Wilson (2009). This wide-scale clustering may generate non-random PN-to-KC connectivity, since a KC dendritic arbor centered on a cluster of boutons arising from a given PN subtype may oversample that subtype. The above-chance rate of multi-claw input to KCs from a tightly bundled subset of three PN subtypes observed in adult *Drosophila* (Gruntman and Turner, 2013) is consistent with this possibility. Our EM-based reconstruction demonstrated tight clustering of these same subtypes (Figure 5A). However, available EM data in larva and LM data in adult *Drosophila* (Caron et al., 2013, Eichler et al., 2017) indicate random sampling of olfactory input, which has become an assumption in many models of odor representation (Dasgupta et al., 2017, Litwin-Kumar et al., 2017, Stevens, 2015; but see Koulakov et al., 2011, Pehlevan et al., 2017). Although in the larval dataset KCs were comprehensively reconstructed at the EM level, adults have ∼20x more KCs as well as additional KC subtypes (Aso et al., 2014, Eichler et al., 2017, Lin et al., 2007), and may therefore have a different network structure. The adult LM data were pooled across many animals and, therefore, could be confounded by inter-individual variability. More comprehensive mapping of intra-animal PN-to-KC connectivity in the whole-brain dataset will help resolve whether or not olfactory input to KCs in adult *Drosophila* is random.

Kenyon cell dendrites in the MB calyx and MB-CP2 axons contain a mixture of synaptic input and output. Mixed input/output (I/O) neurites have previously been found in multiple regions (Meinertzhagen and O’Neil, 1991, Rybak et al., 2016, Schürmann, 2016, Takemura et al., 2017a, Tobin et al., 2017; data not shown), and may turn out to be pervasive in the fly brain. Mixed I/O arrangements can be physiologically and behaviorally important; for example, in the antennal lobe, inhibitory inputs into olfactory receptor neuron axons are known to mediate gain control and temporal sharpening (Olsen and Wilson, 2008, Raccuglia et al., 2016, Root et al., 2008). The whole-brain EM dataset supports the identification of the cell types present at mixed I/O synapses, an otherwise challenging class of information to obtain.

*Drosophila* exhibits a wide range of complex sensory- and memory-guided behaviors. The algorithms underlying behavior are implemented by neuronal circuits, and neuronal circuits are defined, in large part (though not entirely; Bargmann and Marder, 2013), by the synaptic connectivity between neurons. Therefore, connectome maps are necessary to analyze neuronal circuits at the implementation level (Marr, 1982) and could aid in the inference of underlying algorithms as well. The dataset we share here should help establish a structural scaffold for future models of circuit function in the fly and enable comparisons of circuit architecture across species.

## STAR★Methods

### Key Resources Table

**Table:** 

REAGENT or RESOURCE	SOURCE	IDENTIFIER
**Deposited Data**		

The full adult fly brain electron microscopy image dataset	This paper	http://www.temca2data.org/bigtiles.html
The full adult fly brain electron microscopy image dataset at virtualflybrain.org	This paper	https://catmaid-fafb.virtualflybrain.org

**Experimental Models: Organisms/Strains**		

*Drosophila* [iso] Canton-S G1 females	Janelia Research Campus, Howard Hughes Medical Institute (HHMI)	N/A

**Software and Algorithms**		

TEMCA2 control software	This paper	N/A
ATPS control software	This paper	N/A
CATMAID: source code	Saalfeld et al., 2009, Schneider-Mizell et al., 2016	https://github.com/catmaid/CATMAID
CATMAID: user documentation	Saalfeld et al., 2009, Schneider-Mizell et al., 2016	http://catmaid.readthedocs.io/en/stable/user.html
CATMAID: administrator documentation	Saalfeld et al., 2009, Schneider-Mizell et al., 2016	http://catmaid.readthedocs.io/en/stable/administrator.html
CATMAID: developer documentation	Saalfeld et al., 2009, Schneider-Mizell et al., 2016	http://catmaid.readthedocs.io/en/stable/developer.html
TrakEM2	Cardona et al., 2012	https://imagej.net/TrakEM2; https://github.com/trakem2/TrakEM2
mpicbg transformation library and feature-based point match generation	Saalfeld et al., 2010	https://github.com/axtimwalde/mpicbg
Section order correction	Hanslovsky et al., 2017	https://github.com/saalfeldlab/section-sort
Cross-correlation-based point match generation and slab alignment	This paper	https://github.com/billkarsh/Alignment_Projects/blob/master/00_DOC/method_overview.md
Global volume alignment	This paper	https://github.com/khaledkhairy/EM_aligner
Renderer	This paper	https://github.com/saalfeldlab/render
Image tile intensity correction (Distributed Gradient-Domain Processing)	Kazhdan et al., 2010; this paper	http://www.cs.jhu.edu/∼misha/Code/DMG/
Signal-to-noise ratio quantification	This paper	https://github.com/bocklab/temca2data/tree/master/SNR
Neuron skeleton analysis code	This paper	https://github.com/bocklab/temca2data/tree/master/geometry_analysis
Electron-light microscopy tools (ELM)	Bogovic et al., 2016	https://github.com/saalfeldlab/elm
elmr	This paper	https://github.com/jefferis/elmr
NBLAST	Costa et al., 2016	https://github.com/jefferislab/nat.nblast
NBLAST online	Costa et al., 2016	http://jefferislab.org/si/nblast
R neuroanatomy toolbox	Jefferis et al., 2007, Costa et al., 2016	https://github.com/jefferis/nat
R flycircuit	Costa et al., 2016	https://github.com/jefferis/flycircuit
nat.flybrains	Manton et al., 2014	https://github.com/jefferislab/nat.flybrains
rcatmaid	This paper	https://github.com/jefferis/rcatmaid
CATMAID-to-Blender	Schlegel et al., 2016	https://github.com/schlegelp/CATMAID-to-Blender

**Other**		

CATMAID-in-a-box: installation documentation	This paper	http://www.temca2data.org/data.html
CATMAID-in-a-box: workstation	Dell	Dell Precision 5720 with 16 GB of RAM, i5-7600 processor
CATMAID-in-a-box: operating system	Dell, pre-installed	Ubuntu 16.04
CATMAID-in-a-box: CATMAID with Docker	Saalfeld et al., 2009	http://catmaid.readthedocs.io/en/stable/docker.html
CATMAID-in-a-box: installation code	This paper	https://www.github.com/bocklab/temca2-catmaid
CATMAID-in-a-box: RAID storage device	Oyen Digital	Part#3R5-EB3-M
CATMAID-in-a-box: Hard disk drive	Seagate	ST4000NM0035
Precision piezo motor	Physik Instruments	Cat#N301K151
Vacuum chamber extension to FEI T-12 TEM	This paper	N/A
Sample support grids	This paper	N/A
TEMCA2 unit camera	Fairchild	SciMOS 2051 Model F2
TEMCA2 scintillator, 10 mg/cm2 P43 on 5 μm Mylar	Grant Scientific	Custom part
Fast Stage	This paper	N/A
Automated Transport and Positioning System (ATPS)	This paper	N/A

### Contact for Reagent and Resource Sharing

Further information and requests for resources and reagents should be directed to and will be fulfilled by the Lead Contact, Davi D. Bock (bockd@janelia.hhmi.org). The following are available to nonprofit organizations upon request: control software for TEMCA2 and ATPS; CAD model files for the Automated Transport and Positioning System (ATPS), vacuum chamber extension to the FEI T-12 TEM, sample support grids, and the Fast Stage.

### Experimental Model and Subject Details

Multiple brains of 7-day-old [iso] *w*^*1118*^ x [iso] Canton-S G1 adult female flies were screened and one was picked for EM imaging. By this age, development has completed and flies are sexually mature (Ashburner et al., 2005). Females and males were maintained in the same vials.

### Method Details

#### Sample Preparation

Brains from 7-day-old adult [iso] *w*^*1118*^ x [iso] Canton-S G1 flies were dissected in cold fly saline. The dissected brains were fixed with 2% glutaraldehyde in 0.1M sodium cacodylate for 1 h at 4°C, followed by 1 h at room temperature (RT). Following aldehyde fixation, the brains were rinsed 6 × 5 min with sodium cacodylate buffer at RT, 3 × 10 min incubations in 0.02M 3-amino-1,2,4-triazole (A-TRA) (Sigma-Aldrich) in sodium cacodylate, the last on ice, followed by post-fixation with 1% OsO_4_ in sodium cacodylate containing 0.1M A-TRA for 90 min on ice (Van Emburg and De Bruijn, 1984). The brains were then rinsed with cold sodium cacodylate buffer, allowed to warm to RT followed by deionized or Milli-Q water at RT before being stained *en bloc* with 7.5% uranyl acetate (Figure S3A) in water overnight at 4°C. Following *en bloc* staining, brains were rinsed with water at RT and then dehydrated in an ascending ethanol series to 100% ethanol, followed by 100% propylene oxide. Samples were infiltrated with EmBed 812 resin using propylene oxide to resin ratios of 2:1 and 1:2 for 30 min each followed by 2 × 1 h incubations in 100% resin and a third 100% resin incubation overnight. Samples were flat embedded between Teflon-coated glass slides and allowed to harden for 24 h at 65-70°C. Samples were subsequently screened for whole-brain sectioning by X-ray tomography (Figure S3B) using an Xradia XRM-510 X-ray microscope (subsequently acquired by Zeiss). Samples without obvious surface defects due to dissection, or internal defects were re-embedded *in silico*n rubber molds for sectioning.

#### Sample Supports, Ultramicrotomy, and Post-staining

Custom patented 3 mm bar-coded grids made from 100 μm thick copper beryllium with a 2 × 1 mm slot, a unique serial identifier in human readable and 2D barcode form and with fiducial markers were used to collect sections (Figure S3H) (Price and Bock, 2016a). Schematics and vendor information for the custom grids are available to non-profit research organizations upon request. Grids were prepared for picking up sections by first applying a silver/gold-color film of Pioloform (Pioloform FN, Ted Pella catalog #19244) followed by a ∼8 nm layer of carbon. The Pioloform film was made thicker than normal to provide enhanced sample stability under the higher beam current necessary for rapid imaging. To prepare the Pioloform film, a 600 μL aliquot of 2.05% Pioloform in dichloroethane was applied to an ethanol and hydrofluoric acid cleaned glass microscope slide (Gold Seal, Ted Pella catalog #260210) via spin coating using a Laurell WS400B-6NPP/Lite spin coater. After applying the Pioloform solution, the slide was spun for 1.4 s with a target speed of 8,000 rpm and an acceleration index of 255. The film was released from the slide by scribing the edges of the slide with a diamond scribe and slowly submerging the glass slide at a shallow angle into a large dish of water. The film remains floating on the surface of the water and cleaned grids were then carefully placed, bar code side down, onto the film. The film and grids were subsequently picked up from above on a 1 × 3 inch slotted and anodized aluminum slide. The anodized surface provided a stable and reusable surface from which the grids could be cut from the surrounding support film using a heated tungsten filament. Grids were loaded onto custom stainless steel plates for carbon coating.

Carbon coating was carried out in a Denton Explorer 14 high vacuum evaporator equipped with oil diffusion pump, liquid nitrogen cold trap, and a film thickness monitor using carbon rods (Ted Pella catalog #62-132). The carbon rods were de-gassed at sub-evaporation currents (8-14 A) prior to and immediately following sample loading. The coating plate was held at a 90-degree angle to the source at a distance of 10 cm during evaporation. Following a vacuum recovery period, the carbon rods were de-gassed and warmed at sub- to near-evaporation currents (8-16 A). To avoid overheating the films, carbon was evaporated in a series of cycles (in our hands, each cycle was stopped when the deposition rate reached −0.5 Å/sec and resumed when the deposition rate returned to 0 Å/sec). Vacuum levels prior to evaporation were ∼5 × 10^−8^ torr or better. Evaporation was carried out at 22 ± 1 A. Carbon evaporation was halted at an indicated thickness of 70 to 80 Å and final thickness assessed after a 5 min cool down period. Unsuccessfully prepared films displayed a relaxation of the film tension when held close to water (Figure S3C), whereas successfully prepared grid films remained perfectly flat and taut when held within ∼1 mm of a water surface (Figure S3D). Grid batches in which the tested coatings did not remain flat were rejected.

Serial sections of the brain were cut with a Leica UC-6 ultramicrotome at a thickness of 35-40 nm, with periodic retrimming of the block face. In total, 7,062 sections were necessary to encompass the whole brain. Total sectioning time was ∼3 weeks. Typically, three serial sections were collected on each custom bar-coded grid, thus ∼2400 grids were used for the 7,062 sections.

Following sectioning, grids were stained in 3% aqueous uranyl acetate for 20 min followed by Sato’s lead (Sato, 1968) for 5 min, with ddH_2_O washes after each staining step. To facilitate the staining of ∼2,400 grids, a custom Plexiglass staining device with slots to hold 100 grids at a time, loosely based on the Hiraoka (1972) device, was used.

#### Electron Microscopy

Two types of acquisition systems were used to image the whole fly brain series, both of which used FEI Tecnai Spirit BioTWIN TEMs. The first, the TEMCA2 system (Figure S2A), was equipped with a custom single-axis Fast Stage (Figure S2B; patent pending) (Price and Bock, 2016a, Price and Bock, 2016b), vacuum extension, scintillator (5 μm Mylar on a support ring 9 ^5^/_8_ inches in diameter, coated with 10 mg fine-grained P43/cm^2^; Grant Scientific), and four Fairchild SciMOS 2051 Model F2 5.5 megapixel cameras (2560 × 2160 pixel sensor size) configured in a 2 × 2 array (Figure S3E). The second type of system was an Automated Transport and Positioning System (ATPS) (Figures S2E, S2F, and S3I; Videos S2 and S3) (Price and Bock, 2016a, Price and Bock, 2016b), which was equipped with a custom scintillator (6 mg fine-grain P43/cm2; Grant Scientific), and a single Fairchild SciMOS camera. In both systems, 4:1 minifying C-lenses (AMT Imaging) were mounted on the SciMOS cameras. These systems were previously described in abstract form (Robinson et al., 2016). Schematics and model files for the Fast Stage and ATPS are available to non-profit research organizations upon request.

##### TEMCA2 Fast Stage

The Fast Stage (Figure S2B; Video S1) of the TEMCA2 system has a single high-speed axis of motion. It interfaces seamlessly with the commercial FEI CompuStage goniometer, which provides the other degrees of freedom necessary to position a sample in the TEM. The Fast Stage sample holder is connected to a drive rod, which passes through a custom rolling-element bearing, vacuum sealing bellows, and a rolling-element damper. The drive rod is connected to a slide-mounted encoder which provides nanometer-resolution positional feedback. It is moved linearly by a precision piezo motor (Physik Instrument cat N301K151). The custom rolling-element tip bearing provides rigid lateral support to the drive rod within the outer drive rod tube, while minimizing axial friction required to move the driven mass of the system. The custom rolling-element dampers kill vibrations of the drive rod induced by the pulsed motion of the piezo motor during moves, reducing settle time. Without these dampers, the drive rod would vibrate for hundreds of milliseconds under the pulsed motion of a move, rendering the system unusable. With the dampers, 8-24 μm moves are reliably achieved where all vibrations are damped to less than 5 nm in less than 50 ms (Figure S2C). The miniature vacuum bellows isolates the specimen-holding region of the device from atmospheric pressure of the operating environment. By locating the vacuum bellows just behind the O-ring in an FEI style holder, the volume needed to be evacuated after sample insertion is minimized, allowing samples to be exchanged in the same amount of time as a conventional holder (Price and Bock, 2016b).

##### Image acquisition

For TEMCA2-imaged samples, a 16.2 nm/pixel pre-bake mosaic was acquired at 60 ms exposure time to pre-irradiate the sample and reduce specimen warping and shrinkage under high-dose acquisition. The 16.2 nm/pixel mosaics were used to specify ROIs for 4 nm/pixel mosaic acquisition. The 4 nm/pixel mosaics were acquired at 35 ms exposure times. Frames were analyzed for drift in real time and four frames with less than 16 nm frame-to-frame drift were translated into pixel-level alignment, summed, intensity corrected, and saved. Mosaics were acquired in a boustrophedonic fashion column by column running down the long axis of the 2 × 1 mm slot across the three sections such that use of the fast, piezo-driven stage axis was maximized during acquisition while slower CompuStage moves were minimized (Figure S2D). Due to non-overlapping FOVs on TEMCA2, a two-step approach was used where a small stage displacement (∼1900 pixels, or 7.6 μm) filled the gap between the FOVs, and was followed by a large displacement (∼5500 pixels, or 22.0 μm) moving to a completely fresh FOV; this schema was used on both x and y axes with x and y steps being slightly different (5550/1950 and 5450/1850, respectively, big step/small step, in pixels). Accurate calibration of pixels per micron is essential for converting pixel distances into physical distances and allows for pixel distances to be kept constant while the conversion factor was varied depending on the indicated magnification of the microscope. Image acquisition on the TEMCA2 system was performed at an indicated scope magnification of 2900x, while the single camera ATPS equipped system operated at 4800x indicated magnification. The longer vacuum extension of the TEMCA2 system enlarged the projected image by ∼1.7x, resulting in ∼4 nm/pixel for both systems.

##### ATPS grid handling

To better support automatic sample handling, we made custom 100 μm-thick beryllium-copper sample support grids, each etched with unique identifier numbers and spatial fiducial marks (Figure S3H) to guide machine vision-based pick-and-place software for grid exchange (Price and Bock, 2017).

The ATPS grid positioning system (Figures S2E, S2F, S3K, and S3M) mounts to an accessory port on the TEM and is a complete replacement for the FEI CompuStage goniometer and specimen holder, providing all required degrees of freedom to position a specimen within the TEM column, and incorporating a vacuum system allowing for sample exchange under vacuum. High-speed single-axis motion is supported by the same drive mechanisms used in the Fast Stage. Other axes of motion are provided by piezo-driven linear and electromagnetic rotary motors (Figure S2F). The rotational angle of the sample can be changed by placing the sample grid on a rotary pre-aligner, rotating to the desired angle, and picking the sample back up again in the gripper (Video S3). The machine vision system enabling automated handling of samples in the ATPS recorded continuous video while operating, providing visual confirmation of proper operation and an invaluable debugging tool in the event of handling errors. To enable a continuous video stream as well as high dynamic range images suitable for image processing, the acquisition stream automatically adjusts image gain and exposure time for the required regime. These changes can be seen in Video S3.

Samples are organized in the ATPS as follows. The ATPS holds a magazine (Price and Bock, 2015) containing eight cassettes, and each cassette holds 64 sample grids for a total magazine capacity of 512 sample grids (Figure S3J). The ATPS affords random access to the individual grids, which can be retrieved, oriented, loaded into the TEM, imaged, and reliably returned to their proper address in the ATPS. In the event of issues during grid exchange, the exchange is automatically halted, and the grids re-stowed. Re-stowed grids were removed from the ATPS and imaged manually with a Fast Stage on a TEMCA2. The ATPS imaged samples in a two-pass routine where grids were returned to cassettes between acquiring pre-bake mosaics and 4 nm/pixel mosaics. The interval between imaging steps allows for the designation of ROIs for 4 nm/pixel imaging. To ensure that ROIs were accurately acquired, the ATPS found the center of the grid slot every time a grid was loaded into the TEM column. This center point was used to align ROIs and correct for small differences in grid orientation resulting from the two-pass workflow. The ATPS system employed a single point autofocus routine at the center of each section to determine focus for each ROI acquired. High-speed generation of mosaics necessitates high electron dose rate at the sample (typically ∼180x the dose rate required for a 2 s exposure on Kodak 4489 film at 120 kV) to saturate the sensor wells within the short interval (35 ms in our case, versus ∼1 - 2 s typical integration time). Pre-irradiation images of the grids were used to subdivide the samples into three ROI classes: (1) included areas sufficiently free of substrate damage and contaminants to sustain imaging at the highest beam currents; (2) excluded areas to be masked out of the dataset entirely; (3) borderline areas of usable but lower quality to be imaged at 1/10 intensity.

##### TEMCA2 and ATPS system control

Software control of the TEMCA2 and ATPS systems was written in LabVIEW (National Instruments). Wrapper software to interface the Fairchild SciMOS cameras with LabVIEW was written in C. Hardware triggers were used to interleave stage motion with camera frame buffer acquisition. Each camera was read out by a dedicated analysis workstation (Dell), or ‘acquisition node’, connected via 10 Gb Ethernet to a central ‘control node’ which managed hardware triggering, stage control, ROI specification, mosaic preview, and user interface for hardware control. Low-latency TEM hardware control (such as beam blanking, valve operation, CompuStage control, magnification and focus adjustments, and electron beam diameter) was achieved by direct communication between LabVIEW software and the FEI dynamic-link library (DLL) files supporting the FEI Tecnai scripting environment, through the DLLs’ component object model (COM) interfaces.

Acquisition nodes measured translational drift between successive image frames in near real-time, using the National Instruments image analysis package. If image frame drift exceeded a user-specified threshold, the image frame was discarded and additional frames were acquired until the requested number was acquired or until a user-specified timeout was exceeded. Each acquisition node allocated three tiers of memory buffer to the image processing pipeline, to allow real-time acquisition to continue unimpeded, regardless of variations in CPU load, operating system memory management, disk performance, or network throughput. In the first tier, raw image frames were processed for drift estimation. In the second tier, sets of image frames were translated (to correct for small translations by the sample stage), summed, normalized to a background image of the scintillator, and histogram-adjusted. In the third tier, the summed and normalized images were written to disk. As images exited each of these buffers, memory was recycled so new images could be acquired and processed. Due to the rapid rate of data acquisition, multiple storage servers, each connected via 10 Gb Ethernet, were written to in round-robin fashion. Each server contained two RAID-6 volumes, and up to four servers were deployed in parallel during data acquisition. If a RAID-6 volume or a server went offline, images were written to other volumes in the available set. Solid state drives were installed in each acquisition node to allow an acquisition to complete in the event of total network failure during acquisition. This infrastructure was capable of supporting sustained output from the two TEMCAs and the ATPS. No data were lost due to storage or network issues during acquisition of the whole-brain EM volume.

ATPS control software was substantially similar to the TEMCA2 software except that it also controlled the ATPS hardware. ATPS-specific functionality included machine vision-guided pick-and-place and pre-alignment of sample grids, automatic focus of the TEM, and ROI relocation across grid picks. We also developed a user interface to let the operator define the sequence of imaging steps to be performed as well as accompanying microscope parameters for each step. All software for control of the SciMOS cameras, TEMCA2 systems, and the ATPS is available to non-profit research organizations upon request.

##### Section loss

Nearly all (99.83%) targeted serial section data were successfully acquired. In total 12 sections were lost prior to full resolution imaging (Figure S5K). Four sections (4192, 4353, 4951, 6462)) were lost during sectioning; and two grids, each containing three serial sections (3595-3597 and 6883-6885)), were found to have ruptured support films after post-sectioning staining but prior to EM imaging. Two sections (4474, 3715)) were blown when electrons hit artifacts prior to full resolution imaging of the section. Sections with debris or cracks in the support film were imaged in two rounds: a high-dose, high-throughput round, excluding potentially fragile areas of a section; and a subsequent low-dose, slow exposure round, of the fragile region only. Twenty-five sections in nine grids ruptured toward the end of the second-round imaging when the low-dose electron beam hit artifacts. However, a majority (if not the entirety) of the section was already successfully imaged. In this case, although the sections were successfully imaged, the support film rupture precludes future re-imaging of these sections.

The largest serial section gaps were of three adjacent sections (sections 3595-3597 and 6883-6885; the result of two support film ruptures while grids were in storage, awaiting imaging). No other adjacent sections were lost. Image acquisition of the whole-brain began before the ATPS was complete, so most (∼83%) of the sections were acquired by the TEMCA2 systems, and the remainder was acquired by the ATPS. No sections were lost or damaged due to ATPS or Fast Stage malfunction.

##### Acquiring the whole brain

The data were acquired over a period of ∼16 calendar months. The TEMCA2 systems were staffed by a single microscopist, working normal business hours; a second microscopist staffed the ATPS once it came online. Eighty-three percent of imaged sections were acquired with a TEMCA2 system (4.3 million Fast Stage moves), while the ATPS was still in development, and 17% of imaged sections were acquired by the ATPS (3.5 million grid positioning system moves; ∼6,800 machine vision-guided steps to pick, pre-align, and re-stow each grid). Eighty-two percent of ATPS grid exchanges were successful; 14% were automatically halted and the grids re-stowed, usually due to variations in the manual placement of grids in the ATPS cassettes or inhomogeneities in the support film; and 4% required manual control of the ATPS for re-stowing. Re-stowed grids were removed from the ATPS and imaged manually with a Fast Stage on a TEMCA2.

##### Acquisition rates

The TEMCA2 *per-section* acquisition rate is 50 MPix/s, which does not include overhead such as sample exchange time and definition of ROIs. Since the ATPS only has one camera instead of a 2 × 2 array, its per-section acquisition rate is ∼1/4 that of a TEMCA2, or 12.5 MPix/s. With overhead included, our effective *per-grid* throughput for TEMCA2 was 27 MPix/s. The TEMCA2 overhead was ∼10 min per grid, whereas the ATPS overhead was ∼5 min per grid due to autonomous sample exchange and predefined ROIs. Therefore the per-grid throughput for the ATPS was 8 MPix/s, slightly faster than 1/4 of the TEMCA2 per-grid throughput. If an ATPS was mounted on a TEMCA2 system it could achieve another 4-fold throughput increase, for ∼32 MPix/s per-grid throughput. Microscopist supervision was needed for most of the whole-brain acquisition. Assuming a 2000-hour work year (50 weeks at 40 hours per week) for ∼16 months, it took ∼2,666 microscopist hours to acquire the ∼106 TB whole-brain dataset. Estimated *operator* throughput, derived from the total microscopist hours, is therefore ∼11 MPix/s.

The ATPS grid exchange failure rate (18%) had minimal impact on its daily productivity for two reasons. For most exchange failures, the problematic grid was re-stowed automatically and imaging of the next grid began. Grids that were not successfully acquired by the ATPS were unloaded from the ATPS cassettes and imaged manually on a TEMCA2.

For comparison of acquisition rates between the TEMCA2 and other techniques, *per-section* throughput was used for ssTEM and TEMCA2. For ssTEM, TEMCA, and TEMCA2, step and settle times for sample motion during mosaic acquisition were included. Data rates for ssTEM were calculated based on image acquisition parameters used by one of us (RF) during acquisition of the dataset described in Takemura et al. (2013). Data rates for FIB-SEM, SBEM, and ATUM-SEM were calculated from published values (Briggman and Bock, 2012, Kasthuri et al., 2015).

#### Volume Reconstruction

Volume reconstruction includes stitching images from a single thin section into a coherent mosaic and aligning mosaics across all sections. Stitching and alignment of ssTEM images into a traceable volume must overcome artifacts and distortions introduced in sample preparation, serial sectioning, pickup, staining, and imaging of samples. During sectioning, ultrathin sections can be torn, misfolded, or lost completely. EM-dense debris may be introduced during sectioning and/or post-staining of thin sections, obscuring the sample during imaging (Figures S5D and S5E). This debris can charge and heat in the electron beam, putting the support film at risk of rupturing. The sample can also deform during high-intensity imaging. These challenges have largely been addressed for smaller EM volumes (Saalfeld et al., 2012), but the scale of the whole fly brain required the development of a cluster-backed software alignment pipeline to successfully assemble the dataset. Importantly, the new alignment pipeline also enabled projection of traced neuron reconstructions across different versions of the aligned dataset. This allowed tracing to begin before acquisition of the entire series was complete, and preserving tracing done in previous versions of the aligned volume.

##### Data migration

Camera images were written in a round-robin fashion across multiple high-speed storage servers. Mosaics selected for inclusion into the final reconstructed volume were copied to a centrally managed distributed file system at Janelia Research Campus offering high-throughput connectivity to the computational cluster as well as off-site backups. All images were checksum verified after file copy operations.

##### Calibration mosaics

In our TEMCA2 system, we operated with a wider FOV than a conventional TEM which comes at the cost of individual images showing significant non-linear distortion. This distortion is the accumulation of camera lens-distortion, variation in camera mounting, and warping in the electron beam path. We compensated for this distortion using the lens-correction method available in TrakEM2 (Cardona et al., 2012, Kaynig et al., 2010) followed by affine normalization between all distortion models. For each individual camera, we imaged a 3 × 3 mosaic of redundantly (60%) overlapping tiles of a neuropil compartment in one of our sample grids. This mosaic was then used to estimate a non-linear distortion correction model in TrakEM2. To compensate for the remaining affine distortion (scale and shear) of each of these camera models, we imaged a large reference mosaic in the neuropil compartment of three reference sections (to account for accidental section loss) that we then jointly aligned with TrakEM2. This way, we obtained a globally consistent camera calibration model for each individual camera. We repeated the calibration step each time an imaging system was adjusted.

##### Stitching and alignment

The image acquisition process provides partially overlapping images that are assumed to cover each entire section. Image mosaics need to be stitched within each z section plane, as well as aligned across z to produce a seamless volume. The stitching and alignment process includes: (1) matching point-pairs within mosaics, (2) matching point-pairs across mosaics, (3) registering high-dose/low-dose images, (4) section order correction, and (5) solving the volume.

Details of the methods and documentation of actively used code are available at:

https://github.com/billkarsh/Alignment_Projects/blob/master/00_DOC/method_overview.md

https://github.com/billkarsh/Alignment_Projects/blob/master/00_DOC/ptest_reference.md

https://github.com/khaledkhairy/EM_aligner

##### Matching point-pairs within mosaics

The first step of the stitching process is to match putatively identical content between pairs of overlapping images; those matched point-pairs are stored in a table. Matching was done within each of the serial sample sections (z-layers), considered independently of any other sections. Two neighboring images would match essentially perfectly except for very slight differential beam heating.

TEM stage coordinates provide useful guesses about which pairs of images have overlaps worth characterizing, as well as the expected relative transform between pair members that we can use to constrain content matching. For each prospective pair of images we first perform coarse matching using normalized fast fourier transform (FFT)-based cross-correlation to obtain a best rigid transform between them: relative rotation and xy-translation. The expected constraint transform enters as a mask describing a disc of preferred xy-translations within the correlation image.

The coarse transform between image A and B is then refined using a deformable mesh as follows. Within the overlap region of A and B, the A-pixels remain at fixed coordinates. For the B-image pixels, we erect a mesh of triangles and each of the B-pixel coordinates within are translated into barycentric coordinates (functions of the triangle vertices) which are variables. The normalized cross-correlation between A and B can now be expressed as a function of mesh vertex coordinates. A gradient descent process is used to find vertex positions that optimize correlation.

The reported point-pairs linking A to B are derived from the triangles of the mesh. Image-point A is defined as the centroid of a given mesh triangle prior to optimization. Its corresponding B-image point is obtained by calculating the affine transform that takes the triangle to its optimized counterpart, and applying that to the A-centroid.

##### Matching point-pairs across mosaics

Since the layers are nominally 40 nm thick and neural processes propagate through tissue at all possible angles, content in adjacent layers is grossly similar but isn’t a precise match. Nevertheless, content-based matching as described above for same-layer image pairs (FFTs followed by deformable mesh optimization) works very well if combined again with expected pair-pair transforms for which we have high confidence.

First we match whole layers to each other: For each layer, individually, we collect the reported in-layer point matches and solve for its set of affine transform parameters that register that layer’s 2D images to form a so-called montage. These data are used to render the layer at a reduced scale (∼20X) to an image that we call the ‘montage scape’. Scale reduction allows the problem to fit comfortably in RAM, reduces computation time, and most importantly, emphasizes larger size tissue features such as large neurites running parallel to the z axis, which vary slowly as a function of Z. Each pair of montage scapes is matched by FFT cross-correlation at a series of angles and the best correspondence is determined. This is followed by manual inspection using TrakEM2 (Cardona et al., 2012) to verify this rough alignment.

##### Registering high-dose/low-dose images

High-dose/low-dose imaging (see Electron Microscopy) of robust and fragile areas of a section, respectively, could be stitched together seamlessly (Figures S5D–S5F). The majority of high-dose/low-dose sections are acquired during a single session, without the sample being removed from the microscope. Therefore, a reliable first guess for relative positions of these layer patches is usually provided. Generally, high-dose/low-dose sections are registered in a process that takes advantage of components of the general registration pipeline above. Montages of individual acquisitions are generated and their point-matches stored. All montages sharing the same z-value (i.e., the high-dose/low-dose group of sections), together with reference neighbor ‘sandwich’ sections are treated as a set of sections that are roughly aligned to each other as if they were all separate sections. This rough alignment is used to determine potential overlap of high-dose and low-dose areas. Tile-pairs are determined and their point-matches calculated and stored. Finally, all point-matches (within-layer, across high-dose/low-dose patches, and cross-layer to neighboring reference sections) are used to solve a linear system to determine transformation parameters for a seamless registration.

##### Section order correction

We implemented a fully automated procedure for whole-layer matching. Image features are extracted from section montages (Saalfeld et al., 2012), and point-correspondences are determined for all pairs of sections within a range of expected ordering mistakes (in our case within 100 sections). During alignment of the volume, ∼250 sections were found to be misordered. We then used the number of point-correspondences between two sections as a surrogate for their inverse relative distance and identified the shortest possible path to visit all sections, resulting in an ordered series (Hanslovsky et al., 2017). Then, a regularized linear system is solved to calculate an affine transformation for each section that roughly aligns the volume.

With all of the layers now coarsely aligned, we subdivide each layer into an array of ‘blocks’ (∼10 × 10 neighborhoods of camera images). We again step angles and calculate FFT cross-correlation, this time on pairs of corresponding blocks to find the best block-block cross-layer transforms. As a result we know which images within the blocks pair with each other and what their relative transform ought to be. Again, we subdivide each image into local regions, estimate point correspondences using FFT-based cross-correlation, and collect these correspondences in a database.

##### Solving the volume

With the full set of point-pairs tabulated, each image is typically connected to several of its neighbors. We then construct a system of equations requiring that, under the sought affine parameter set that defines each image transformation, point-pairs should map to the same global point in the reconstructed volume. To avoid spurious deformation and volume shrinkage, the equation system is regularized to a roughly aligned volume. This roughly aligned volume depends on individual montages that were in turn regularized to a rigid model approximation that is independently estimated. The full system constitutes a large linear sparse matrix problem, whose solution provides the globally optimal transformation for all images simultaneously.

##### Sources of error

*Wrong (low-quality) point-pairs*. These may occur due to the self-similarity of nominally good quality neural EM images. Errors are even more likely in tissue regions that are substantially devoid of neurons or texture, such as the lumen of the esophagus, or along the outer boundary of the sample where tissue is sparse or even absent from several camera images. To address this error we employ (a) auxiliary contextual information about the likely transform between any two images that constrains matching derived from local image content alone, and (b) we impose a strict point-matching filter using Random Sample Consensus (RANSAC) (Fischler and Bolles, 1981) to separate true correspondences that behave consistently with respect to an affine transform up to a maximal correspondence displacement (Saalfeld et al., 2012).

*Missing point-matches*. In some cases tissue damage, contamination or folds within a section lead to a lack of point-matches in a smaller region within the volume. This is most prominent when searching for point-matches across z. We address this issue by extending the point-match search beyond immediate neighbor sections.

##### Image Intensity Correction

Variations in section thickness, electron beam etching, or deposition of contaminants from post-staining or section pickup create intra-mosaic variations in camera image intensity. For ease of tracing, these variations were corrected (Figures S5G–S5J) using a scalable implementation of an existing algorithm (Distributed Gradient-Domain Processing) (Kazhdan et al., 2010).

During iterative volume reconstruction, gradient-domain processing is used to remove seams in two dimensions. A target gradient field is constructed by computing the gradient field of the input mosaic and zeroing out seam-crossing gradients. Then, a least-squares system is solved to find the new image whose gradients best fit the target field. In addition, low-frequency modulation is removed by computing the windowed average of adjacent mosaics and replacing the low-frequency components of an input mosaic with the low-frequency components of the average. We anticipate that future work will allow 3D processing of the whole-brain image volume (Kazhdan et al., 2015), reducing or eliminating section-to-section variations in intensity.

##### Projection of tracing across alignments

With each new alignment, the CATMAID PostgreSQL database containing all neuronal skeleton coordinates (Schneider-Mizell et al., 2016) is dumped to retrieve their ‘world’ coordinates (coordinates representing their physical location in the brain). Each of these world coordinates is then inversely transformed using the Renderer service (see Stack management and relational database) to a set of ‘local’ coordinates detailing the source tile visible at that location and the relative location within. The local coordinates are projected back into world coordinates using the new alignment’s transformations. The updated coordinates are then applied to a new copy of the database.

##### Stack management and relational database

We created a relational database for storing and querying metadata associated with the thousands of image mosaics and millions of acquired images. We use SQL Server 2012 for our production system and SQLite for development. Metadata required for downstream processing included: paths to image data (with checksums), stage coordinates, ROIs associated with nominal section numbers, ordering of sections and microscope configurations with associated calibrations. The input for the alignment process – a stack – can be generated with a single SQL query joining the majority of tables. The result is a list of images with their layer (z), stage coordinates (x,y), and camera configuration (for associating the correct lens correction model).

The alignment process of the approximately 21 million images and associated projection of already-traced skeletons between alignment iterations is computationally expensive. To manage this we developed the Renderer toolkit (https://github.com/saalfeldlab/render), a set of image stack management tools and RESTful HTTP web services now in use in multiple additional projects. Renderer was designed in order to handle large -scale (hundreds of millions) individual records efficiently while supporting large-scale concurrent access for the stitching, section order analysis, skeleton mapping and intensity correction. Briefly, Renderer is able to quickly materialize (i.e., render) modified images for a set of transformation parameters using the mpicbg transformation library (https://github.com/axtimwalde/mpicbg). The use of the mpicbg library allows simple conversion between the Renderer database (a MongoDB instance) and TrakEM2 projects. For large-scale rendering and coordinate mapping, we used Java stand-alone and Spark framework clients to allow it to be processed in bulk on a cluster.

##### Assessment of alignment quality

Reconstructed neuron skeletons typically recapitulate the natural tortuosity of neuronal arbors, but can be distorted artificially by section misalignments. Large abrupt changes in the coordinates of skeleton nodes often indicate section misalignments. To evaluate the alignment quality of the data, smoothness of reconstructed neurons was assessed. The reconstructed skeletons of all PNs, KCs, and the APL were convolved with a Gaussian kernel (σ = 12 μm). The σ was chosen to eliminate large abrupt changes in the skeletons. The distance between each node of the original skeletons and the smoothed skeletons was computed, and the mean distance for all skeleton nodes in each section was then calculated. The absolute difference between the mean distance in each section and that in the preceding section was computed, which we call the displacement (Figure S5L). The median and the 95% percentile of the displacements are 0.09 μm and 0.57 μm, respectively.

#### Neuron Tracing

Neuron reconstructions are based on manual skeleton tracing of neuronal arbors and annotation of synapses from image stacks in CATMAID (http://www.catmaid.org) as described in Schneider-Mizell et al. (2016). All neurons included in analyses are reconstructed by at least two team members, an initial tracer and a subsequent proofreader who corroborates the tracer’s work. In the event that any tracer or proofreader encounters ambiguous features (neural processes or synapses that are not identifiable with high confidence), they consult other tracers and proofreaders to determine the validity of said features, climbing the experience ladder up to expert tracers as needed. If any feature remains ambiguous after scrutiny by an expert tracer, then said feature is not included in the neural reconstruction and/or flagged to be excluded from analyses. During the proofreading phase, the proofreader and tracer iteratively consult each other until each neuron is deemed complete per the specific tracing protocol to which it belongs. An assignment of completion does not necessarily entail that an entire neuron’s processes and synapses have been reconstructed (see Tracing to classification and Tracing to completion). We traced 114 PNs, the APL, two MB-C1s, MB-CP1, and two MB-CP2 neurons to classification (120 neurons in total). We also traced the calyx sub-arbors of the 15 KCs to completion, and their remaining sub-arbors to morphological, but not synaptic, completion. The total cable length of the neurons above is 206.6 mm.

The criteria to identify a chemical synapse include at least three of the four following features, with the first as an absolute requirement: 1) an active zone with vesicles; 2) presynaptic specializations such as a ribbon or T-bar with or without a platform; 3) synaptic clefts; 4) postsynaptic membrane specializations such as postsynaptic densities (PSDs). In flies, PSDs are variable, clearer at postsynaptic sites of KCs in a microglomerulus but often subtle, unclear, or absent in other atypical synaptic contacts (Prokop and Meinertzhagen, 2006). In the absence of clear PSDs, we marked all cells with membranes that have unobstructed access to a clearly visible synaptic cleft as postsynaptic. We did not attempt to identify electrical synapses (gap junctions), since they are unlikely to be resolved at the resolution of this dataset.

Very rarely, aberrant neurons were found in the dataset. For example, two PNs with a piece of fused cell membrane were discovered in our tracing. It is unknown what factors might cause this, but cell membrane pathologies resultant from EM fixation protocols have been observed (Kopek et al., 2017). Overall, however, the ultrastructural quality of the whole brain was excellent.

##### Tracing to classification

Often only reconstruction of backbone (e.g., microtubule-containing backbone neurites) (Schneider-Mizell et al., 2016) or gross morphology is needed to classify a neuron based on expert identification or NBLAST-based neuron searching against an existing LM dataset. If either approach fails to find a match (as in the case of MB-CP2 in our study), the neuron may be deemed a new cell type. Neurons traced to classification are at a minimum skeletonized, with or without synapses, to the point at which their gross morphologies (or backbone skeletons) unambiguously recapitulate that observed by LM for a given cell class, or are unambiguously deemed as a new cell type not previously observed in LM databases from NBLAST neuron morphology search and/or multiple experts.

##### Tracing to completion

All steps for tracing to classification were completed. Additionally, for morphological completion every identifiable neurite is traced, and for pre- or postsynaptic completion every identifiable presynaptic release site or postsynaptic density, respectively, on the neuron is annotated. Some neurons were traced to morphological or synaptic completion only in certain brain regions (e.g., PNs were traced to morphological, but not synaptic, completion in the MB calyx).

##### Quantification of tracing speed

We reconstructed 114 olfactory PNs to classification and 15 KCs to morphological, but not synaptic completion, for a total cable length of 153.4 mm and 11.6 mm, respectively. The mean PN cable length was 1.35 mm and the mean KC cable length was 0.78 mm. The PN total construction time was 1,272 h and the KC total construction time was 166 h, with mean per cell construction times of 11.2 h and 11.1 h. To calculate tracing speed we divided the mean cable length produced by the sum of the mean time required for both manual tracing and proofreading for all 114 PNs and all 15 KCs reported in this manuscript. Tracing and proofreading timestamps for all additions, deletions, modifications, and proofreading of skeleton node events were recorded into a database and can be queried via a history widget in the CATMAID tracing environment. Construction time was measured as active bouts of tracing and proofreading. Active bouts were defined as temporal windows during which tracing and proofreading timestamps were continuously applied to a neuron by each user with no larger than a 3 min pause. Any 3 min pause or longer introduced a gap between active bouts, and the next active bout began at the next timestamped event of that neuron. The duration of active bouts for all users for each neuron was then summed to determine total tracing and proofreading time.

##### Validation of tracing accuracy

To test the reproducibility of tracing in the whole-brain EM dataset, three independent teams each comprising two members, one tracer and one proofreader, each reconstructed one KC sub-arbor to completion in tracing environments blinded to each other (Figure S6). The KC sub-arbor included the soma, all arbors in the MB calyx, and a proximal portion of the MB pedunculus (Figure S6A). In the fly brain, microtubule-free neurites (twigs) as fine as 40 nm in diameter tend to extend for short distances before joining larger, microtubule-containing backbone neurites (Schneider-Mizell et al., 2016). KC claws are good examples of twigs, whereas their dendritic trunks and the axonal neurite leaving the MB calyx are larger-diameter backbones. In the tracing phase, the tracer had access to the proofreader for consult and verification. During the proofreading phase the proofreader had access to the tracer for consult and verification. The neuronal arbors and associated synapses reconstructed by each team were essentially identical for both twigs and backbones. An expert validated all sites of disagreement between the three teams to determine a ‘gold-standard’ skeleton of the KC. PN-to-KC claw inputs with high synapse counts were detected in all three reconstructions (Figure S6B). Consistent with a tracing approach biased toward false negatives rather than false positives, only one input with low synapse count was missed by one of the tracing teams (Figure S6, red asterisks).

We re-implemented a skeleton-to-skeleton agreement measurement (Helmstaedter et al., 2011) to locate the sites of discrepancy between the skeletons of each of the three teams and the gold-standard skeleton, respectively. All skeletons were resampled with an interval of 80 nm (Schneider-Mizell et al., 2016), and nodes from each team skeleton were compared with nodes from the gold-standard skeleton. Each team node was considered to be consistent with a gold-standard node if the team node was within 800 nm of a gold-standard node. Thresholds smaller than 800 nm resulted in correct reconstructions of lower order neurites to be inconsistent. We visually inspected the inconsistent nodes to determine the number of errors. All errors were false negatives. The length of missed skeletons for each error was measured (Figure S6A). Error rates differ significantly between microtubule-containing backbones and microtubule-free twigs (Schneider-Mizell et al., 2016). In our tracings, the shortest path from soma to MB pedunculus of the KC (spine), and the first order branches of the spine (arms) that give rise to the claws were classified as backbone as these neurites contain microtubules. The remaining higher order branches (e.g., claws) are considered twigs. We found no errors for 167 μm of backbone skeletons, and the twig (287 μm) mean error rate was 37.9 μm/error. The total mean error rate for the gold standard KC skeleton was 60.8 μm/error (mean number of disagreements was 7.3 across a neuron 454 μm in extent). Our error rates were compared with those from two previous studies. In mammalian retina (Helmstaedter et al., 2011), the total mean error rate for five-fold independent tracing of a single neuron was 83.4 μm/error (mean number of disagreements was 7.2 across a neuron 600 μm in extent); backbone and twig error rates were not reported separately. In *Drosophila* larva, the mean error rate for an iteratively traced skeleton was 375.8 μm for backbone (three errors) and 16.2 μm for twigs (95 errors) (Schneider-Mizell et al., 2016). In total 1127 μm of backbone cable length and 1,539 μm of twig cable length were traced. Therefore, with 98 errors across a neuron 2,666 μm in extent, the total mean error rate was 27.2 μm/error in the larva.

##### Tracing of PNs

Three protocols were used to reconstruct PNs on the right side of the brain. First, putative PN boutons presynaptic to all claws of ∼200 KCs traced from a separate ongoing effort were seeded and traced to classification. Second, a seed section at the posterolateral bend of the mALT, proximal to MB calyx, was selected and all neurons not found via the first protocol were traced directly toward the calyx. Neurons that innervated MB calyx were traced to classification, whereas those that bypassed calyx were halted. Third, a thorough visual survey of the MB calyx was conducted to ensure that all microglomerular structures had been identified and the untraced boutons within these microglomeruli were seeded with single skeleton nodes then traced to classification.

We focused our classification efforts on uniglomerular olfactory PNs that provide input to the MB main calyx. Additional PNs were discovered but excluded from PN counts per subtype (Figure 4E) if they bypass calyx. The expert identification of PN subtypes was based on gross morphology. Key diagnostic features included the somatic locations, projection tracts, and distribution of arbors in the AL (the glomerulus innervated by each PN), MB calyx, and LH. Classification of olfactory glomeruli in AL followed that of Grabe et al. (2015), with a few exceptions: we did not attempt to classify VP2, VP3, and VP4 because they are either non-olfactory or multimodal (Enjin et al., 2016, Grabe et al., 2015); our reconstructions indicated clear independent domains for VC3l and VC3m glomeruli (consistent with Chou et al., 2010, Silbering et al., 2011); and VC3l and VC5 could not be disambiguated. PNs arising from these two glomeruli were therefore left unclassified, but future tracing of the neurites from neurons in surrounding glomeruli may clarify their identities. Following Grabe et al. (2015) and Yu et al. (2010), VM6 and VP1 were combined into a single glomerulus due to morphological ambiguities, which we label as VM6 in this work. Reconstruction of ORNs, multiglomerular/multimodal PNs, and tracing of PN dendrites to completion, will likely clarify remaining ambiguities in glomerular classification.

Axonal boutons of PNs in the MB calyx were identified by varicosities containing arrays of presynaptic active zones, each of which was apposed to many postsynaptic processes (Figure S1A). Skeleton nodes at the varicosity/intervaricosity borders were tagged as ‘bouton borders’ such that they contained all synapses inside each varicosity.

Of the 578 microglomerular boutons in MB main calyx, 497 arose from olfactory PNs (86%, from 114 PNs). Of these, 16 boutons (3%) arose from six olfactory multiglomerular PNs. The other inputs include 17 boutons (3%) arising from MB-CP2; nine boutons from other PNs (2%, arising from four neurons), traveling either via tracts alternative to the mALT (eight boutons from three PNs) or from the subesophageal region (one bouton from one putative PN; data not shown); 55 boutons (9%) from the other 10 unidentified (presumably non-olfactory, e.g., thermosensory, hygrosensory) multiglomerular or uniglomerular PNs.

##### Tracing MB-CP2 to classification

The two MB-CP2 neurons were traced to classification. In this process, many second order branches within each neuropil compartment were traced. To obtain a representative sample of the synapses in the right hemisphere, we selected several third order branches in each brain region, traced them to their terminal processes (the highest order of branching within that region), and annotated all synapses on these neurites. We identified its synaptic partners in cases where those neurons had already been traced for other purposes (e.g., previously reconstructed KCs). Most cell types in the fly brain have symmetric contralateral equivalents (Aso et al., 2014, Chiang et al., 2011, Jenett et al., 2012). Therefore we also reconstructed the contralateral MB-CP2 neuron and its synapses to assess the symmetry of its morphology and connectivity, a previously used technique to validate the reconstruction accuracy of known and previously unidentified cell types in the *Drosophila* larval EM volume (Eichler et al., 2017, Ohyama et al., 2015, Schneider-Mizell et al., 2016) (Figure 6A). Fewer processes and synapses were traced and annotated for the MB-CP2 neuron in the left hemisphere than for the MB-CP2 neuron in the right hemisphere.

##### Kenyon cells and their postsynaptic partners in the MB calyx

Three KCs from each of the five KC classes that innervate MB main calyx (γ, αβc, αβs, α’β’m, and α’β’ap) were randomly selected from a larger set of several hundred KCs already traced to classification as part of a separate, ongoing study. Only presynaptic sites in the neuropil region of the MB calyx were included. Synapses in MB pedunculus and lobes were not reconstructed. All postsynaptic partners at each presynaptic site were enumerated and traced to classification.

The 14 previously known cell types that could potentially be postsynaptic partners of KCs in MB main calyx were enumerated by a literature search. They are: the octopaminergic ventral paired median neurons (OA-VPM3 and OA-VPM5), the octopaminergic ventral unpaired median neuron (OA-VUMa2); the putatively GABAergic glutamic acid decarboxylase interneurons (GAD1-GAD4), MB-C1, MB-C2; the contralaterally projecting serotonin-immunoreactive deutocerebral (CSD) neuron; the dopaminergic posterior lateral protocerebum neuron (PPL2ab); the MB calyx MBON MB-CP1; the lateral horn leucokinergic (LHLK) neuron; and the APL (Aso et al., 2014, Burke et al., 2012, Busch et al., 2009, Chen et al., 2012, de Haro et al., 2010, Mao and Davis, 2009, Roy et al., 2007, Tanaka et al., 2008).

#### Neuronal Informatics

##### Electron-Light Microscopy registration tools (ELM)

The ELM tool provides a user interface to interactively define a thin plate spline 3D warp field between an LM dataset and the whole-brain EM dataset by manually specifying corresponding landmark points. It was built on top of the BigWarp Fiji plugin (Bogovic et al., 2016), which in turn was built on top of the BigDataViewer plugin (Pietzsch et al., 2015) for Fiji (Schindelin et al., 2012). The ELM software is aware of standard compartment boundary models available for the template fly brains and provides hotkeys to view the labels for these compartments; to go between coordinates in ELM and the EM dataset as viewed in CATMAID; and to go from a CATMAID URL to the corresponding point in ELM. Code for ELM is available at https://github.com/saalfeldlab/elm. This software can be used to map individual LM-imaged neurons that have been registered to a template brain into the EM whole brain.

An LM image volume of a whole fly brain in which random fluorophore combinations are expressed in a subset of PNs (MultiColor FlpOut) (Nern et al., 2015) was kindly provided by Yoshino Aso and colleagues (Y. Aso, personal communication). The image volume was registered to the JFRC2013 template brain. Using the JFRC2013-to-EM volume transformation defined above the PNs were then projected into the coordinate space of the EM whole brain.

##### Transforming data between EM and LM templates: elmr

The elmr software tool is a package (https://github.com/jefferis/elmr) written in R (http://www.r-project.org) to facilitate bidirectional transfer of 3D data between the EM whole-brain dataset and LM template brains. This package reproduces the thin plate spline transformation that maps between the whole-brain EM volume and the JFRC2013 template brain (Aso et al., 2014) from the landmark pairs exported by the ELM tool described in the previous section. Using an approach described by Manton et al. (2014), 3D data can then be transformed into other light level template brains used by the fly neurobiology community, including the template (JFRC2, a.k.a. JFRC2010) (Jenett et al., 2012) used for data on the virtualflybrain.org site. This process depends on an additional R package (nat.flybrains, https://github.com/jefferislab/nat.flybrains) that provides non-rigid registrations defined with the CMTK image registration toolkit (https://www.nitrc.org/projects/cmtk). This and all additional R packages used for subsequent analysis are automatically installed as dependencies of elmr. Inverse transformation is also supported, enabling data registered to a light level template to be projected into the space of the whole-brain EM volume.

##### Surface models of neuropil compartments

Previously defined surface models of the whole fly brain and several brain regions (Ito et al., 2014, Jenett et al., 2012, Manton et al., 2014), based on the same template brain as the virtualfybrain.org project (https://github.com/VirtualFlyBrain/DrosAdultBRAINdomains), were transformed (Manton et al., 2014) to the EM volume using elmr. The AL glomerulus meshes were generated in Blender (https://www.blender.org/) from EM-reconstructed skeletons of PN dendrites and ORN termini retrieved with the CATMAID-Blender interface (Schlegel et al., 2016).

##### Remote deployment for tracing in the whole brain (“CATMAID-in-a-box”)

We provide a preconfigured tracing environment including the CATMAID tracing software, the reconstructed neurons presented in this manuscript, and scripts to download the entire EM volume from the temca2data.org website (http://www.temca2data.org/). The CATMAID software has built-in functionality for importing and exporting neuron skeletons, which facilitates sharing of traced neurons across laboratories.

Groups wishing to use CATMAID-in-a-box will need a mid-range Linux or Mac workstation capable of running Docker Community Edition and 12 TB of disk space to store the EM volume. We tested a Dell Precision 5720 (16 GB of RAM with a quad-core 3.5 GHz Intel i5-7600 processor) and a commodity external RAID-5 enclosure configured with five 4 TB hard disk drives. Full details for suggested configurations are maintained on the temca2data.org website.

CATMAID (and its backing PostgreSQL database) are distributed as Docker containers to facilitate installation without complicated dependencies. The full 12 TB image volume is downloaded over the Internet. The provided script to download data will checkpoint its status to allow resumption in the event of network interruption. Full step-by-step installation instructions are provided on the temca2data.org website.

### Quantification and Statistical Analysis

#### Comparison of SNR between Volume EM Datasets

Determining the SNR of biological images is in general a subjective task, due to its variance under non-linear transformations (Erdogmus et al., 2004). As users of this data will likely care about biological structures, the determination of SNR should account for this, considering only the level of signal of these structures and not of things such as staining or cutting artifacts. The problem of SNR determination has been thoroughly treated in the case of super-resolution imaging where these ambiguities don’t exist (Lambert and Waters, 2017; see also Supplementary Note 1 in Li et al., 2015), but as yet there are no universally accepted, automated techniques to calculate the SNR in individual images where signal is dense in both spatial and frequency spaces, such as EM data of brain.

We present two measures of SNR here, an automated measure which avoids user biases, but can include some signal in noise and background calculations (feature-based SNR), and a simple technique which gives more precise SNRs but is prone to bias (the cell membrane technique) which we use to verify the feature-based SNR calculation. We apply these techniques to a range of publicly available data in order to evaluate the TEMCA2 method. Sample images from each dataset are shown in Figure S4A.

We assume all methods are shot-noise limited. For comparison purposes SNR values are normalized to the TEMCA2 voxel size (4 × 4 x 40 nm) by the square root of the source data’s voxel size. In both the feature-based SNR and the cell membrane SNR measures, we assume that noise is additive (and is independent of the magnitude of the signal) and symmetric. Such an assumption is likely false (e.g., electron shot noise is Poissonian and not symmetric at low numbers), however, such impacts are likely small based on manual examination of images and we assume the impacts of such an assumption are the same for all techniques. Such assumptions may fail, however, at very low signals where Charge-coupled device (CCD) and shot noise dominates, or at high signals, where processes such as non-linearity in CCD absorption become important.

##### Feature-based SNRs

Fundamentally, an SNR calculation of an image involves a calculation of the background level, the variation in this background level (which is assumed to be due to noise) and the calculation of the difference between the ROI and this background. Detecting what these regions are provides a challenge in EM data where images may not have clear background regions and where noise is contributed to through sample preparation.

In order to measure the SNR we assumed that in any given image, the structures of interest provide the majority of features above the noise. That is, most structures present are biological in nature, rather than artifacts of sample preparation. Therefore with this assumption, it can be further assumed that key-points detected by feature detection algorithms will disproportionately fall on the ROI.

Given that cells and structures therein tend to be ‘blobby’ due to hydrostatic processes (Jiang and Sun, 2013), we used a blob-detection algorithm (which compares areas of interest, *cf.* edge or corner detection) to identify areas of interest. We used the SURF algorithm (Bay et al., 2008).

Following the above, the variation in intensity of an image, *I*, in the local region of many feature points is likely to be mostly due to signal, and the variation in intensity nearby few (or no) feature points will be dominated by noise. The determination of such regions was done by generating an array of equal size to the original image and for each element, setting it to one if there is a feature in the corresponding element of the image. This array was then convolved with a Gaussian of width *n*, where *n* was chosen to maximize the SNR in a random selection of five images from each sample in an effort to avoid bias between samples.

To select a region dominated by noise we then shuffled this array before sorting it (to avoid biases in sorting algorithms) and took the lowest point. We then blocked out a region 2*n* square and resorted the array nine more times (for a total of 10 selected regions), forming the set of points *p*_low_. We likewise performed a selection for the points of maximum variation (*p*_high_). See Figure S4B for an illustration of the entire process.

To determine the level of noise, we first generated a copy of the image to which a three pixel median filter had been applied. We then subtracted this median image from the original to generate a noise dominated image, *I’*. At each of the minimum feature points (where noise is most dominant); the standard deviation of this image was taken over a three pixel square neighborhood. The level of noise, *N*, was then calculated as the mean of these standard deviations, i.e.,

(1) $$N=\langle \sigma \left[I^{'}\left(p_{low}\pm 1\right)\right]\rangle .$$

The background level of the image, *B*, was determined by taking the mean of these noise-dominated regions (again taking a mean over the three pixel neighborhood), following on from the assumption of symmetric noise, giving

(2) $$B=\langle \langle I\left(p_{low}\pm 1\right)\rangle \rangle .\;$$

The level of signal was then taken to be the mean of the (absolute) difference of the mean of these three pixel neighborhoods around *p*_high_, and the background. This resulted in the SNR being given by

(3) $$S/N=\frac{\langle \left|I\left(p_{high}\pm 1\right)-B\right|\rangle }{N}.$$

As most images lack large areas that consist of only resin, this simple background selection is not perfect. Therefore, the SNRs generated should be considered lower limits in most cases. We show the SNR as a function of the acquisition rate for a variety of EM techniques in Figure 2G.

To validate the feature-based method, we examined the effect of varying resolutions and confirmed that SNR changed in the expected manner for the additive Gaussian noise model. The feature-based method works reasonably well when combining voxels producing SNRs within 20% (1.5 dB) of the expected based on additive Gaussian noise (Figure S4C), although the ATUM-SEM data of Kasthuri et al. (2015) increases by more than others, a possible sign of their voxels (3 × 3 nm in x-y) under-sampling biological features. This method produces the expected increase when scaling down images producing equivalent normalized SNRs (Figure S4C). Increasing the size of images also increases the SNR, but this is due to the generation of new pixels with similar values to old ones inside the regions considered for noise due to the fact that creation of these new pixels functions as a pseudo-low pass filter. As expected this measure reports larger SNR values when Gaussian blurring is applied (as noise is disproportionately removed when a low pass filter is applied) (Figure S4C). In images generated by super resolution techniques therefore, this method may be inappropriate and should be modified to, for example, use distance based regions rather than pixel based regions.

##### Cell membrane SNR

Although the feature-based SNR measure avoids many human biases in the selection of regions used to calculate background and signal levels, it unfortunately can often incorporate biological structure (our signal of interest) into these calculations.

We therefore introduced a complementary measure to compare the SNR of biological EM data and verified that the feature-based SNR calculation is valid. At its heart, this is simply a comparison between the signal level at a cell membrane and the background nearby, taken at multiple points within an image.

This was achieved by a user creating a line inside a random 100 × 100 pixel region which contains only resin and, ideally nearby, a line which covers only a stained cell membrane. Pixels along these lines were considered to be background or signal, respectively. After selection of a background and signal line within each region, another 100 × 100 pixel region was chosen, until 20 lines in total (10 background lines, 10 signal lines) were selected, skipping a region if there was not a suitable location to select both.

The noise was considered to be the standard deviation of the pixel intensities across all background intensities, and the background level the mean. The signal value was considered to be a mean of the signal pixels.

We show an example of the selection process in Figure S4D. Signal-to-noise ratios found by this method (Figure S4E), were within 10% of those found via the feature-based method, suggesting the former may be used for a fast, bias-free comparison between methods.

#### Quantification of Artifacts

In order to examine the quality of the dataset, an assessment of missing and degraded tiles was undertaken. Using the losslessly compressed tiles, we examined each 1024 × 1024 pixel region from which the CATMAID image tile pyramid is built, and checked if this region was first, present, and second, if it was degraded due to an artifact in the data. We plot the total number of tiles and number of degraded tiles per section in Figure S5K. A section was considered to have missing tiles, if its tile count was 5% below that of the median of the neighboring five sections on each side. Qualitatively, this was the threshold at which biology is noticeably impacted and not arising due to a tighter ROI around the brain. Within the core region of the brain (sections 2000-6000), 3% of sections had tile loss above this threshold, with 1% of all sections having extensive tile loss exceeding 20%. In addition to these missing tiles, a tile was considered degraded if the standard deviation of its grayscale histogram either exceeded 40, or was below 20 and had a mean grayscale value below 50. These thresholds included small-scale precipitate, folds and intensity correction artifacts in the former and large-scale precipitate errors in the latter. These degraded tiles comprised 0.5% of the entire dataset with 1% of sections having over 5% of degraded tiles.

#### Analysis of Neuronal Geometry

Analysis of neuronal geometry was conducted using custom packages developed in R. We imported skeletons of EM-reconstructed neurons from the CATMAID tracing environment using rcatmaid (https://github.com/jefferis/rcatmaid). Section thickness in CATMAID was specified to be 35 nm; all analyses of skeleton geometry therefore use this value. For qualitative and quantitative comparison with skeletons of LM-reconstructed neurons, the skeletons of EM-reconstructed neurons were transformed into coordinate spaces of various LM template brains using elmr based on landmark pairs defined with ELM (see Electron-Light Microscopy registration tools). The R NeuroAnatomy Toolbox package (nat, https://github.com/jefferis/nat) was used for geometric computations, 3D visualization of both reconstructed neurons and surface models.

##### NBLAST search for PNs

The geometric search tool NBLAST (Costa et al., 2016) was used to aid identifications of PN subtypes (Table S1) by comparing morphological similarity. NBLAST breaks neuron skeletons into segments and computes an NBLAST score based on a geometric comparison of the segments of a given pair of neurons (a query neuron and a target neuron). NBLAST scores range from −1 (completely anticorrelated) to 1 (identical), but for practical purposes, 0 is a natural cutoff. A score of 0 means that the similarity level for segment pairs are, on average, as likely to be from neurons of the same type as they are to be from a random pair of neurons in the database. The EM-reconstructed PNs were transformed into the FlyCircuit template brain space for NBLAST neuron search against the ∼400 LM-reconstructed PNs whose subtypes were previously classified (Chiang et al., 2011, Costa et al., 2016). This is enabled by a single elmr function nblast_fafb. The search functionality is built on the nat.nblast package (https://github.com/jefferislab/nat.nblast) and uses data distributed with the FlyCircuit package (https://github.com/jefferis/flycircuit), both of which are installed with elmr. Only EM-reconstructed PNs whose candidate subtypes exist in the FlyCircuit dataset were used for NBLAST search. The EM-reconstructed skeletons, which typically had many additional fine processes as compared to LM-reconstructed skeletons, were used as the target neuron rather than the query neuron for geometric comparison, in reverse to the conventional NBLAST option. NBLAST search of each EM-reconstructed PN returned the top five hits of LM-reconstructed neurons and their NBLAST scores, which were tabulated to aid expert glomerular identification of PNs. For the VM2 example shown in Figures 3E–3G, the top NBLAST score for the EM-reconstructed VM2 PN was compared with the top NBLAST scores from searching all LM-reconstructed VM2 PNs against each other. Further details of the NBLAST neuron search, the associated LM data, and an online web-app for on-the-fly NBLAST queries are available at http://jefferislab.org/si/nblast.

##### NBLAST clustering and PN dendrogram generation

Pairwise NBLAST scores were computed for all uniglomerular PNs (the nblast_allbyall function in the nat.nblast package at https://github.com/jefferislab/nat.nblast) after transformation into the JFRC2 template brain (Jenett et al., 2012) space using elmr. We used unsupervised hierarchical clustering with Ward’s method based on the NBLAST scores (nat function nhclust).

##### Renderings and analysis of PN arbors in MB calyx

Reconstructed PNs from two LM datasets, one from the FlyCircuit database (Chiang et al., 2011) and the other from Jefferis et al. (2007), were previously registered to a common template brain (Costa et al., 2016), and were used for comparisons with EM-reconstructed PNs reported here. We first determined which PN subtypes had multiple EM- and LM-reconstructed neurons available. For a few PN subtypes, we analyzed EM-reconstructed PNs although no LM reconstructions were available from the dataset, enabling comparison of a more complete set of PN subtypes within the EM modality. The DA3 subtype was excluded because the DA3 PNs from the LM data do not extend collaterals into the MB calyx (Jefferis et al., 2007). For analysis, we then selected a random set of LM skeletons so that we had the same number of LM and EM skeletons for each PN subtype.

For visualizations, LM-reconstructed PNs from the FlyCircuit database were used for Figure 5A, and LM-reconstructed PNs from Jefferis et al. (2007) were used for Figure S7A. Linear interpolation of neighboring skeleton nodes was applied to EM-reconstructed PNs to smooth artifactual spikes in neuron tracings due to registration errors. EM- and LM-reconstructed PNs were resampled with a 1 μm interval to ensure uniform representation of skeletons. For analysis, LM-reconstructed PNs from both the FlyCircuit database and Jefferis et al. (2007) datasets were used. For both visualization and analysis, EM- and LM-reconstructed PNs were transformed onto a common template brain (Costa et al., 2016, Jenett et al., 2012, Manton et al., 2014).

To obtain the subset of PN arbors ramifying in MB calyx for both visualization and analysis, a bounding box defined by the calyx surface mesh (Ito et al., 2014, Manton et al., 2014, Milyaev et al., 2012) in the JFRC2 template brain was used to trim the skeletons of EM- and LM-reconstructed PNs. For visualization in Figure 4C, the boutons of PNs (as defined in Tracing of PNs) in MB calyx were isolated and used to generate bouton volumes by applying a Gaussian smoothing. For analysis of PN arbors in the MB calyx (Figures 5B, 5C, S7B, and S7C), the backbone of each PN was removed so that only the collaterals entering the calyx were included in geometric measurements.

We computed geometric measurements (pairwise distance and NBLAST scores) for only the MB calyx arbors of EM- versus LM-reconstructed homotypic PNs. For each PN subtype, pairwise distances quantified physical co-location of arbors and NBLAST scores quantified morphological similarity for pairs of neurons. To calculate the pairwise distance, we iterated over each node of a query neuron to find the nearest node in a target neuron, measured the Euclidean distances between each pair of nodes, and calculated the mean nearest distances for all nodes in the query neurons (forward distance). When a query neuron has significantly longer arbors than a target neuron, large nearest distances can be introduced by end points in the query neuron’s arbors that are far away from closest nodes of the target neuron’s arbors. To address this issue, we calculated the same mean nearest distance with the query neuron and target neuron in reverse (reverse distance) and picked only the smaller of the forward and reverse distances, which was the pairwise distance. To quantify morphological similarity, NBLAST scores were computed for PN arbors in MB calyx in a similar pairwise manner. The distribution of all pairwise distances and NBLAST scores of homotypic PNs were plotted, and for both measurements the difference between EM and LM population data were analyzed with a Student’s t test.

### Data and Software Availability

All files and videos are available through the following website: http://www.temca2data.org.

### Additional Resources

Access to the full adult fly brain dataset is available at: http://www.temca2data.org.

Analysis code is available at: https://github.com/bocklab/temca2data.

## Supplemental Information

Table S1. Agreement between Expert and NBLAST-Based Identification of PN Subtypes, Related to Figure 4 Abbreviations: N, no NBLAST match found from the top five hits.

Table S2. KC Postsynaptic Partners in the MB Calyx, Related to Figure 7
