# Supplementary material for: A Complete Electron Microscopy Volume of the Brain of Adult Drosophila melanogaster
Source: Cell. 2018 Jul 26;174(3):730–743.e22. doi: 10.1016/j.cell.2018.06.019 (PMC6063995; doi:10.1016/j.cell.2018.06.019)
Supplement: Table S1. Agreement between Expert and NBLAST-Based Identification of PN Subtypes, Related to Figure 4 — Abbreviations: N, no NBLAST match found from the top five hits. [file mmc1.pdf]

Cell, Volume *174*

Supplemental Information

A Complete Electron Microscopy Volume of the Brain of Adult *Drosophila melanogaster*

Zhihao Zheng, J. Scott Lauritzen, Eric Perlman, Camenzind G. Robinson, Matthew Nichols, Daniel Milkie, Omar Torrens, John Price, Corey B. Fisher, Nadiya Sharifi, Steven A. Calle-Schuler, Lucia Kmecova, Iqbal J. Ali, Bill Karsh, Eric T. Trautman, John A. Bogovic, Philipp Hanslovsky, Gregory S.X.E. Jefferis, Michael Kazhdan, Khaled Khairy, Stephan Saalfeld, Richard D. Fetter, and Davi D. Bock

PN skeleton ids	expert identification	match	NBLAST results									
			1st hit		2nd hit		3rd hit		4th hit		5th hit	
			glomerulus	score	glomerulus	score	glomerulus	score	glomerulus	score	glomerulus	score
27295	DA1	1st	DA1	0.697	DA1	0.688	DA1	0.685	DA1	0.683	DA1	0.672
57311	DA1	1st	DA1	0.701	DA1	0.686	DA1	0.682	DA1	0.680	DA1	0.657
57323	DA1	1st	DA1	0.653	DA1	0.635	DA1	0.634	DA1	0.633	DA1	0.626
57353	DA1	1st	DA1	0.656	DA1	0.645	DA1	0.625	DA1	0.618	DA1	0.617
57381	DA1	1st	DA1	0.717	DA1	0.697	DA1	0.693	DA1	0.678	DA1	0.673
61221	DA1	1st	DA1	0.685	DA1	0.684	DA1	0.681	DA1	0.681	DA1	0.678
755022	DA1	1st	DA1	0.635	DA1	0.618	DA1	0.613	DA1	0.605	DA1	0.604
2863104	DA1	1st	DA1	0.657	DA1	0.640	DA1	0.634	DA1	0.633	DA1	0.624
38885	DA2	1st	DA2	0.639	DA2	0.621	DA2	0.619	DA2	0.589	DA2	0.563
53631	DA2	1st	DA2	0.583	DA2	0.555	DA2	0.529	DA2	0.522	DA2	0.513
57418	DA2	1st	DA2	0.635	DA2	0.623	DA2	0.622	DA2	0.592	DA2	0.578
57422	DA2	1st	DA2	0.615	DA2	0.604	DA2	0.602	DA2	0.581	DA2	0.549
1785034	DA2	1st	DA2	0.602	DA2	0.601	DA2	0.596	DA2	0.564	DA2	0.526
65762	DC2	1st	DC2	0.551	DA2	0.537	DA2	0.514	DA2	0.508	DA2	0.498
32399	DC3	1st	DC3	0.577	VA1v	0.480	VA1d	0.459	VA1d	0.449	VA1v	0.446
57241	DC3	1st	DC3	0.513	VA1d	0.428	VA1d	0.400	DA1	0.393	VA1v	0.376
57414	DC3	1st	DC3	0.581	VA1d	0.457	VA1d	0.442	VA1d	0.428	VA1v	0.425
27303	DL1	1st	DL1	0.644	DL1	0.639	DL1	0.622	DL1	0.612	DL1	0.596
1775706	DL1	1st	DL1	0.680	DL1	0.655	DL1	0.645	DL1	0.641	DL1	0.636
30791	DL2d	1st	DL2d	0.670	DL2d	0.652	DL2d	0.646	DL2d	0.644	DL2d	0.641
57333	DL2d	1st	DL2d	0.672	DL2d	0.670	DL2d	0.670	DL2d	0.666	DL2d	0.664
57337	DL2d	1st	DL2d	0.712	DL2d	0.695	DL2d	0.693	DL2d	0.693	DL2d	0.690
57341	DL2d	1st	DL2d	0.704	DL2d	0.701	DL2d	0.700	DL2d	0.682	DL2d	0.682
22422	DL2v	1st	DL2v	0.699	DL2v	0.696	DL2v	0.691	DL2v	0.686	DL2v	0.681
56623	DL2v	1st	DL2v	0.664	DL2v	0.661	DL2v	0.651	DL2v	0.650	DL2v	0.646
61773	DL2v	1st	DL2v	0.690	DL2v	0.683	DL2v	0.682	DL2v	0.676	DL2v	0.673
33903	DL3	1st	DL3	0.673	DA1	0.644	DA1	0.614	DA1	0.608	DA1	0.602
581536	DL3	1st	DL3	0.638	DA1	0.609	DA1	0.605	DA1	0.605	DA1	0.589
23829	DL4	1st	DL4	0.594	DA1	0.574	DL1	0.458	DL1	0.434	VM7	0.275
30891	DM1	1st	DM1	0.671	putative VA4	0.491	DM5	0.463	DM5	0.433	VC2	0.428
22594	DM5	1st	DM5	0.604	DM5	0.599	VM5d	0.533	VM5d	0.510	VM5d	0.509
27611	DM5	1st	DM5	0.587	VM5d	0.580	VM5d	0.561	VM5d	0.561	VM5d	0.557
57307	DM6	1st	DM6	0.601	DM6	0.596	DM6	0.594	DM6	0.579	DM6	0.575
60799	DM6	1st	DM6	0.633	DM6	0.627	DM6	0.600	DM6	0.593	DM6	0.589
68697	DM6	1st	DM6	0.581	DM6	0.577	DM6	0.567	DM6	0.565	DM6	0.565
27048	DP1l	1st	DP1l	0.665	DP1l	0.530	DL2v	0.463	DL2v	0.460	DL2v	0.456
27884	V	1st	V	0.540	V	0.382	V	0.367	VP1	0.331	V	0.225
192547	V	1st	V	0.511	VP1	0.215	V	0.109	V	0.108	DP1l	0.030
36390	VA1d	1st	VA1d	0.631	DC3	0.545	VA1v	0.540	VA1v	0.517	VA1v	0.515
42421	VA1d	1st	VA1d	0.561	DC3	0.532	VA1v	0.499	VA1v	0.497	VA1v	0.491
51080	VA1v	1st	VA1v	0.627	VA1v	0.620	VA1v	0.610	VA1v	0.605	VA1v	0.602
52106	VA1v	1st	VA1v	0.604	VA1v	0.599	VA1v	0.592	VA1v	0.579	VA1v	0.577
55125	VA1v	1st	VA1v	0.614	VA1v	0.585	VA1v	0.585	VA1v	0.577	VA1v	0.574
57246	VA1v	1st	VA1v	0.654	VA1v	0.641	VA1v	0.635	VA1v	0.634	VA1v	0.622
23569	VA4	1st	putative VA4	0.580	VC2	0.528	DM5	0.495	DM5	0.439	DL2v	0.398
32214	VA7m	1st	VA7m	0.600	VA7m	0.568	DA2	0.457	VM1	0.445	DL2d	0.420
36108	VA7m	1st	VA7m	0.634	VA7m	0.629	DL2d	0.470	DL2v	0.463	DL2d	0.460
186573	VA7m	1st	VA7m	0.587	VA7m	0.581	VC2	0.490	DA2	0.458	VM1	0.447
45242	VC2	1st	VC2	0.640	VC2	0.586	DM5	0.483	DM5	0.470	VM1	0.460
22277	VC3m	1st	VC3m	0.605	VC3m	0.589	VC3m	0.584	VC3m	0.584	VC3m	0.576
22744	VC3m	1st	VC3m	0.591	VC3m	0.584	VC3m	0.584	VC3m	0.581	VC3m	0.576
400943	VC3m	1st	VC3m	0.598	VC3m	0.596	VC3m	0.590	VC3m	0.580	VC3m	0.579
37935	VC4	1st	VC4	0.593	VC4	0.592	VC4	0.563	VC4	0.562	VM7	0.500
42927	VC4	1st	VC4	0.600	VC4	0.554	VC4	0.532	VC4	0.512	VM7	0.503
55085	VC4	1st	VC4	0.607	VC4	0.607	VC4	0.568	VC4	0.557	VM5v	0.516
24726	VM1	1st	VM1	0.690	VM1	0.680	VM1	0.679	VM1	0.674	VM1	0.673
775731	VM1	1st	VM1	0.642	VM1	0.631	VM1	0.626	VM1	0.624	VM1	0.583
51886	VM2	1st	VM2	0.639	VM2	0.620	VM2	0.616	VM2	0.603	VM2	0.574
54072	VM2	1st	VM2	0.679	VM2	0.658	VM2	0.657	VM2	0.642	VM2	0.597
23597	VM4	1st	VM4	0.465	VM4	0.432	VC3m	0.414	VC3m	0.385	VC3m	0.377
39139	VM5d	1st	VM5d	0.598	VM5d	0.597	VM5v	0.589	VM5d	0.583	VM5d	0.570
23512	VM5v	1st	VM5v	0.618	VM5v	0.618	VM5v	0.578	VM5v	0.567	VM5v	0.565
30434	VM5v	1st	VM5v	0.596	VM5v	0.587	VM5v	0.581	VM5v	0.562	VM5v	0.552
53671	VM5v	1st	VM5v	0.596	VM5v	0.580	VM5v	0.559	VM5v	0.538	VM5v	0.530
40306	VM7d	1st	VM7	0.654	VM7	0.653	VM7	0.633	VM7	0.632	VM7	0.622
40790	VM7d	1st	VM7	0.605	VM7	0.599	VM7	0.595	VM7	0.595	VM7	0.584
24251	VM7v	1st	VM7	0.537	VM7	0.524	VM7	0.517	VM7	0.503	VM7	0.503
43539	VM7v	1st	VM7	0.541	VM7	0.522	VM7	0.507	VM5d	0.502	VM7	0.499
24622	D	2nd	DL1	0.532	D	0.523	DL1	0.513	DL1	0.509	DL1	0.489
40637	D	2nd	DL1	0.574	D	0.562	DL1	0.537	DL1	0.521	DL1	0.517
40749	DA4l	2nd	DC2	0.531	DA4	0.512	DL4	0.479	DA4	0.476	DA2	0.470
57402	DC2	2nd	DL1	0.515	DC2	0.506	VM7	0.482	DA2	0.476	DL1	0.475
27246	VM3	2nd	putative VA4	0.539	VM3	0.529	VM2	0.520	VM2	0.516	VC4	0.495
35447	VM5d	2nd	VM5v	0.638	VM5d	0.633	VM5v	0.632	VM5v	0.624	VM5v	0.601
49865	VM5d	2nd	VM5v	0.603	VM5d	0.568	VM5d	0.546	VM5d	0.542	VM5d	0.540
67637	DL2v	3rd	DL2d	0.625	DL2d	0.609	DL2v	0.605	DL2d	0.601	DL2d	0.593
62434	VM5d	4th	VM5v	0.635	VM5v	0.625	VM5v	0.613	VM5d	0.603	VM5v	0.599
35246	D	5th	DL1	0.561	DL1	0.559	DL1	0.549	DL1	0.532	D	0.531
58686	DP1m	N	putative VA4	0.471	DM5	0.459	VC2	0.440	DL2v	0.410	DL2d	0.405
41308	VM3	N	VM2	0.478	VC4	0.456	VM2	0.446	VC4	0.438	VC4	0.413

Table S1. Agreement between Expert and NBLAST-based Identification of PN Subtypes. Related to Figure 4.

Abbreviations: N, no NBLAST match found from the top five hits.
